# Correction: Independent re-analysis of alleged mind-matter interaction in double-slit experimental data

**DOI:** 10.1371/journal.pone.0253686

**Published:** 2021-06-17

**Authors:** Nicolas Tremblay

## Notice of republication

After publication of this article [1], the author was made aware that the statistical test used was flawed. More specifically, the trimming procedure used lead to uncontrolled false positives and thus underestimated p-values.

The data were re-analyzed; this affects all figures except the first three, as well as statements made in the Abstract, Methods, Results, and Discussion. A revised version of the article was republished on June 11, 2021, to address this issue. The updated article includes revised versions of all figures except the three first ones, as well as text updates to the Abstract, Methods, Results, and Discussion sections. Please download the article again to view the correct version. The originally published, uncorrected article and the republished, corrected articles are provided here for reference.

The updated results have been reviewed by a statistical reviewer and a member of PLOS ONE’s Editorial Board, who confirmed that the main conclusions of the article are upheld based on the updated dataset and analyses.

For reference, the original article PDF is published in S1 File of this notice. S3 File includes a track changes version of the manuscript that shows all changes made between the original and updated versions of the article.

The author apologizes for the errors in the original publication.

## Supporting information

S1 File Originally published, uncorrected article. (PDF)

S2 File Republished, corrected article. (PDF)

S3 File Track changes version of the manuscript showing changes made between the original and updated publications. (PDF)
