# Supplementary material for: Independent re-analysis of alleged mind-matter interaction in double-slit experimental data
Source: PLoS One. 2019 Feb 7;14(2):e0211511. doi: 10.1371/journal.pone.0211511 (PMC6366771; doi:10.1371/journal.pone.0211511)
Supplement: S1 File — (PDF) [file pone.0211511.s001.pdf]

Supporting information

Let $0 < \alpha < 1$ be a significance level. We illustrate here that false-positives are uncontrolled in the test used in [1] and under control of α in the correct test described in Section 2.3. To do so, consider the following framework:

i/ Create a synthetic set $\mathcal{X}$ by drawing its n iid elements from $\mathcal{N}(0, 1)$, the Gaussian distribution with zero mean and variance 1. n is set to 10^3.

ii/ Generate $N = 5 \times 10^4$ independent realisations of such set $\mathcal{X}$. All N sets thus have a true zero mean by construction.

iii/ Test each set: obtain a p-value per set and, given the significance level α, a rejection decision per set.

We then consider both the probability of type I error estimated by $\hat{p}_{\mathrm{I}} = R/N$, where R counts the number of rejected sets $\mathcal{X}$ (for the given α), as well as the α-quantile p^*_α of the N p-values obtained: the value under which there are αN p-values. If the test used in iii/ is correct, both $\hat{p}_{\mathrm{I}}$ and p^*_α should be very close to α.

Figure 13 compares results obtained with the test published in [1] and the one described in Section 2.3. For both tests, at $q = 0$ and as expected, both indicators $\hat{p}_{\mathrm{I}}$ and p^*_α are at α, as it should be. However, as q increases, the test from [1] deviates from α quite significantly. For instance, for $q = 0.2$, the 0.01-quantile of the computed p-values is 3.5×10^{-4}, almost two orders of magnitude lower than what it should be!

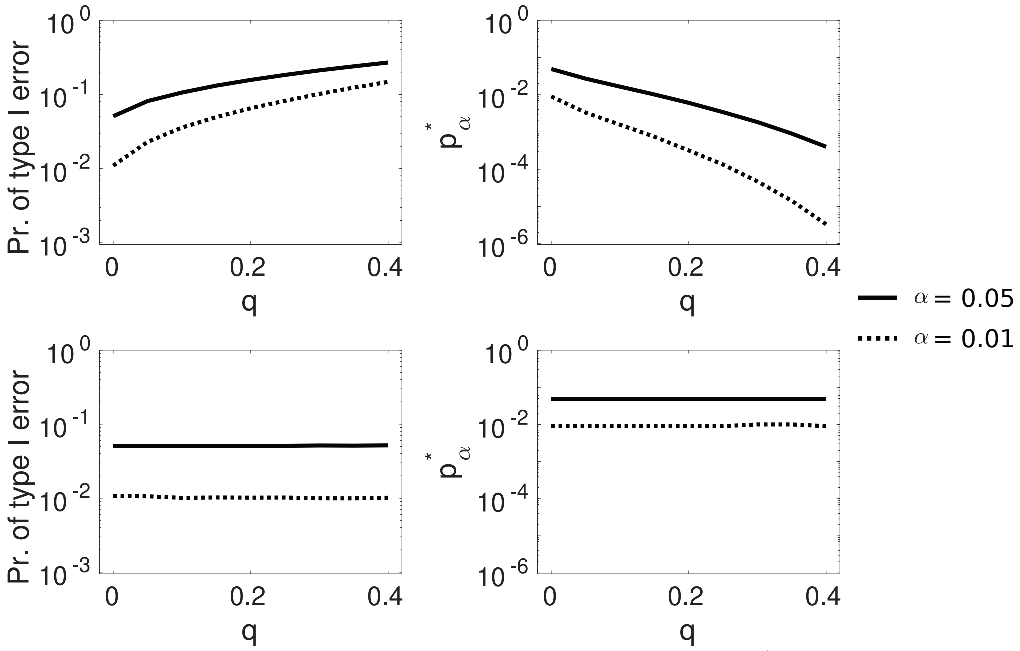

Fig 13. Results obtained on artificial data of zero mean (see the Supporting information for details). Top line: results of the test published in [1]. Bottom line: results of the correct test detailed in Section 2.3. Left: the estimated type I error $\hat{p}_{\mathrm{I}}$ as a function of q (the trimming intensity), for two different values of α. Right: the α-quantiles of the p-values versus q, for two different values of α. Number of bootstrap samples used for both tests: 2000.

On the contrary, in the test used in this paper, and for all values of q, both $\hat{p}_{\mathrm{I}}$ and p^*_α are equal to what is expected from a well-controlled test, namely α.
