# Supplementary material for: Correction: Independent re-analysis of alleged mind-matter interaction in double-slit experimental data
Source: PLoS One. 2021 Jun 17;16(6):e0253686. doi: 10.1371/journal.pone.0253686 (PMC8211243; doi:10.1371/journal.pone.0253686)
Supplement: S1 File — (PDF) [file pone.0253686.s001.pdf]

PLOS | ONE

RESEARCH ARTICLE

Independent re-analysis of alleged mind-matter interaction in double-slit experimental data

Nicolas Tremblay*

CNRS, Univ. Grenoble Alpes, Grenoble INP, GIPSA-lab, Grenoble, France

* nicolas.tremblay@gipsa-lab.grenoble-inp.fr

Check for updates

OPEN ACCESS

Citation: Tremblay N (2019) Independent re-analysis of alleged mind-matter interaction in double-slit experimental data. PLoS ONE 14(2): e0211511. https://doi.org/10.1371/journal.pone.0211511

Editor: L. Krister Shalm, University of Colorado, UNITED STATES

Received: October 19, 2018

Accepted: January 15, 2019

Published: February 7, 2019

Copyright: © 2019 Nicolas Tremblay. This is an open access article distributed under the terms of the Creative Commons Attribution License, which permits unrestricted use, distribution, and reproduction in any medium, provided the original author and source are credited.

Data Availability Statement: The $\sim$ 80 Gb of raw data as well as all Matlab codes used in this paper are publicly available on the Open Science Framework database platform at the address https://osf.io/ywktp/.

Funding: The author received no specific funding for this work.

Competing interests: The author has declared that no competing interests exist.

Abstract

A two year long experimental dataset in which authors of Radin, et al., 2016 claim to find evidence of mind-matter interaction is independently re-analyzed. In this experiment, participants are asked to periodically shift their attention towards or away from a double-slit optical apparatus. Shifts in fringe visibility of the interference pattern are monitored and tested against the common sense null hypothesis that such shifts should not correlate with the participant's attention state. We propose a deeper analysis of the dataset, identifying all the necessary arbitrary pre-analysis choices one needs to make, and carefully assessing the results' robustness regarding these choices. Results are twofold. Firstly, even with a conservative correction for the multiple statistical tests the analysis calls for, we confirm the existence of significant although small anomalies in the direction predicted by the mind-matter interaction hypothesis. On the other hand, and unlike Radin, et al., 2016, we also report significant although even smaller anomalies in the control dataset. This leads us to conclude that this particular dataset does not provide strong evidence of mind-matter interaction, yet certainly contains inexplicable anomalies that should motivate replication attempts in highly controlled environments.

1 Introduction

The hypothesis of a mind-matter interaction, that is, the possibility that human intention may have an impact on matter at a distance, is usually regarded by most physicists as a highly controversial concept. It is nonetheless related to von Neumann's interpretation [2] of the quantum measurement problem, namely that consciousness causes the collapse of the wave function when a quantum system in a superposition of states is observed. Even if this interpretation has been and still is considered by many minds of quantum mechanics [2–4], it is today blatantly disregarded by a majority of physicists [5] partly because it flirts with the overwhelmingly complex mind/body problem. This mysterious link between consciousness and matter appears indeed to have an infinite number of uncontrollable parameters, and therefore does not seem to lend itself to rigorous scientific inquiry. Moreover, von Neumann's interpretation being by all means only one out of many possible interpretations of quantum mechanics [6] –

most of which keep consciousness aside–, physicists generally prefer mathematically controlled objective concepts such as quantum decoherence [7] or Everett's many-worlds interpretation [8]. It is nevertheless well worth reminding that, however strong and heated are personal convictions around this debate, consensus over the quantum measurement problem has not yet been reached [5] and that any attempt to provide empirical information on this matter should be widely welcome.

Along those lines, the experiment first proposed by Ibison and Jeffers in [9] is worthy of interest. Their working hypothesis is that a human subject's attention towards a quantum system may be modeled as an extremely weak measurement of the system, that should in turn imply a proportionally weak but still *measurable* collapse of its wave function. The authors propose to test this hypothesis using one of the simplest quantum apparatus: the double-slit optical interferometer. In this context, it is well-known [10] that if the path taken by photons through the interferometer (called "which-way information") is recorded, then photons behave like particles (they don't interfere), otherwise they behave like waves (they interfere). It has also been verified that the strength of the observed interference pattern is inversely proportional to the amount of which-way information one gathers [11, 12]. Keeping that in mind, and according to the working hypothesis previously stated, a human subject's attention towards a double-slit system, if it really acts as a weak measurement of the which-way information, should very slightly attenuate the interference pattern. Other working hypotheses can be thought of that do not require a gain in which-way information while still accounting for a decrease in fringe visibility. For instance, Pradhan [13] proposes another theoretical background based on a small modification of the Born rule. We will not delve here into the technicalities of these theoretical approaches and refer to the debates and ideas in [13–16] for the interested reader. In this paper, we will essentially concentrate on data and analyze it as carefully as possible to identify anomalies if they exist, regardless of the precise potential mechanism underlying them.

Ibison and Jeffers reported contradictory and inconclusive results from their pioneering experiments [9]. In the last few years, Radin and collaborators [1, 17, 18] reproduced their experiment at a large scale. In their work, the fringe visibility of the interference pattern is monitored while human subjects are asked to periodically shift their attention towards or away from the optical system. In [1], the authors analyze a two-year long experiment with many different subjects, claim to find small but statistically significant shifts of the fringe visibility, and interpret it as evidence of mind matter interaction.

In this paper, we independently re-analyze the dataset presented in [1], providing a bigger picture of the statistical analysis and exploring its robustness with respect to many pre-analysis choices. Note that Baer [19] also proposed an independent re-analysis on half of the data we explored in this paper. On top of providing insights on the full two-year dataset, we follow a more conservative (but nevertheless necessary) line of statistical analyses, systematically correcting multiple testings by Holm-Bonferonni, showing how results vary versus all pre-analysis choices, as well as how robust they are to random subsampling. We argue that no specific fringe should be taken into account alone (nor specific minimum in the case of Baer's analysis). Similarly to both [1] and [19], we report anomalies in the data. Different from both previous studies nevertheless, part of the control data is also found anomalous; which undermines the anomalies found in the human data, and weakens possible conclusions to be drawn from this dataset. Also, in an effort for reproducible research, the ~80 Gb of raw data as well as all Matlab codes used in this paper are publicly available on the Open Science Framework database platform at the address https://osf.io/ywktp/.

The outline of the paper is as follows. We briefly recall the experiment's protocol in Section 2.1, and define the difference in fringe visibility Δv in Section 2.2 as the main statistics we will

focus our analysis on. Sections 2.3 and 2.4 detail the basic statistical tests we perform and preliminary results. The robustness of these results is then assessed in the subsequent Sections 2.5 to 2.9. In Section 2.10 we reduce the dataset to human sessions measured within one hour of a control session to probe any systematic bias due to potential experimental drifts. We then finish our analysis with Section 2.11 where effect sizes are estimated. Section 3 discusses all theses analyses, and Section 4 offers concluding remarks.

2 Materials and methods

2.1 The experiment

The apparatus consists of a laser, a double-slit, and a camera recording the interference pattern; and is located in IONS' laboratory, in Petaluma, California. Details are in [1]. The apparatus is always running, even though the data is only recorded when somebody connects to the system via Internet. A participant to the experiment connects online to the server (accessible through IONS' research website) and receives alternating instructions every 30 seconds, to either "now concentrate" or "now relax". During concentration epochs, the participant's task is to mentally influence the optical system in order to increase a real-time feedback signal, displayed as a dynamic line on the screen. For people who prefer to close their eyes during the experiment, the feedback is also transmitted as a whistling wind tone.

In 2013, the feedback was inversely proportional to a sliding 3-second span average of the fringe visibility: the higher the line, or the higher the pitch of the tone, the lower was the fringe visibility, the closer was the system to "particle-like" behaviour.

In 2014, due to a coding error, the feedback was inversed: the feedback now increased when the fringe visibility *increased*. The participant's task was still to increase the feedback, but this time the higher the line, or the higher the pitch of the tone, the lower was the fringe visibility, the closer was the system to "wave-like" behaviour.

As controls, a Linux machine connects to the server via Internet at regular intervals. The server does not know who it is dealing with: it computes and sends feedback, and records interference data just as it would do for a human participant.

Each session always starts and finishes with a relaxation epoch. A total of 10 concentration and 11 relaxation epochs are recorded per session, which makes the whole session last about 10 minutes and 30 seconds. Some sessions end before all epochs are completed, due to Internet connection issues, or to participants' impatience. One possible bias could come from participants' self-selection: it could be argued that participants with poor results quit the experiment earlier than participants performing well. To avoid this bias, we need to take as many sessions as possible into account. On the other hand, very short sessions do not enable a precise estimation of any measurable difference between the two types of epochs. We decide to keep only sessions containing more than $\tau = 1000$ camera frames, which correspond to sessions approximately completed half-way and containing 8 alternating epochs. We will see in Section 2.7 how the value of τ changes the results.

Given $\tau = 1000$, the dataset is comprised of 3679 sessions in 2013 (2374 of which are controls) and 4976 in 2014 (3363 of which are controls).

2.2 Pre-analysis: From the raw data to difference in fringe visibility

The camera records at 4Hz a line of 3000 pixels, an example of which is shown in Fig 1, where are also displayed the maximum and minimum envelopes (noted env_M and env_m in the following) of the interference pattern computed with cubic spline interpolation between local extrema. Local extrema are automatically detected after a Savitzky-Golay filter of order 2 on a 29-pixel moving window that smooths the interference pattern in order to remove the pixel

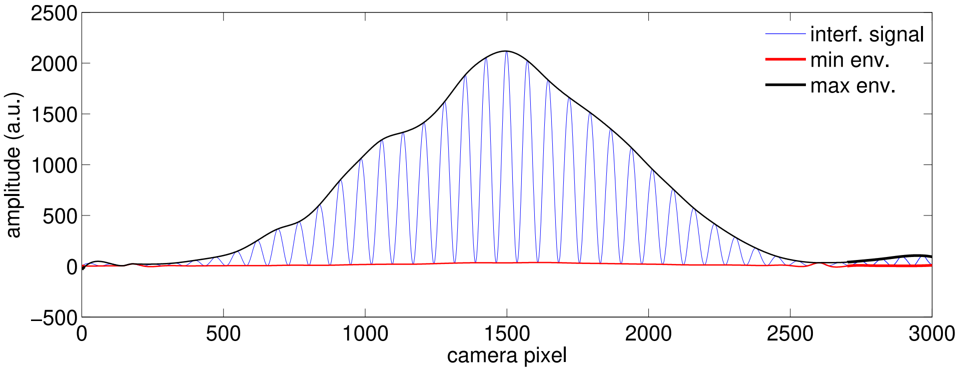

Fig 1. The interference pattern. Example of a camera shot of the interference pattern, along with its two spline interpolated envelopes.

https://doi.org/10.1371/journal.pone.0211511.g001

jitter that appears on some camera frames. We have also tried other smoothing options: same order Savitzky-Golay filters with 39 and 49-pixel window-lengths, as well as simple moving average filters with 20 and 30-pixel window-lengths, with no significant change in the overall results.

For a better signal to noise ratio, we consider the 19 middle fringes of the pattern. Fig 2 shows such a zoom, as well as the fringe visibility function, defined as:

$$fv = \frac{\mathrm{env}_M - \mathrm{env}_m}{\mathrm{env}_M + \mathrm{env}_m}. \tag{1}$$

For each camera frame, we extract one scalar. The choice of this scalar is not straightforward and we will explore different choices throughout the paper. Following the analyses published in [1], we start by concentrating on the average of the fringe visibility around fringe number 9, that is, on the interval represented in Fig 2 between two vertical dashed lines. We will see in Section 2.5 how results change if one considers other fringe numbers, or averages over more than one fringe.

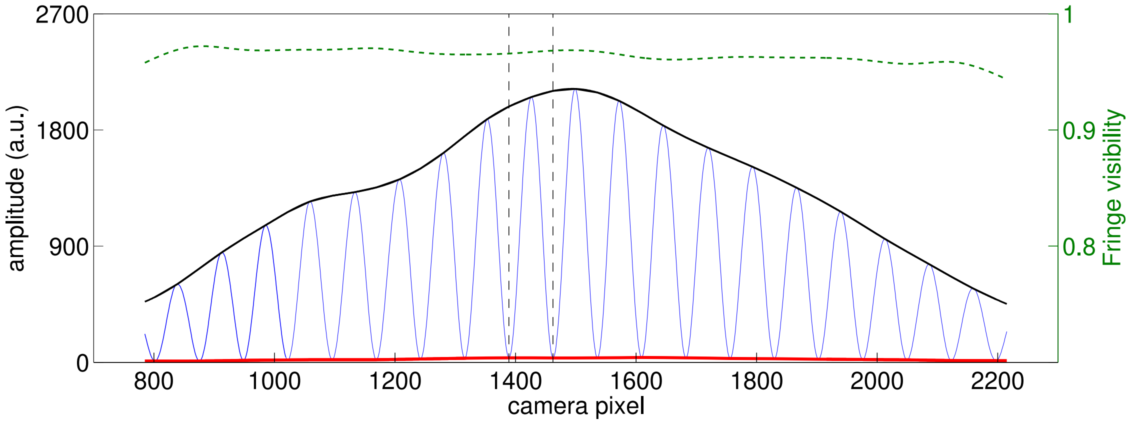

Fig 2. Zoom on the interference pattern. Zoom around the 19 middle fringes of the interference pattern, along with its two interpolated envelopes. The fringe visibility as defined in Eq 1 is shown in dashed green. The two vertical dashed lines represent the interval corresponding to fringe number 9.

https://doi.org/10.1371/journal.pone.0211511.g002

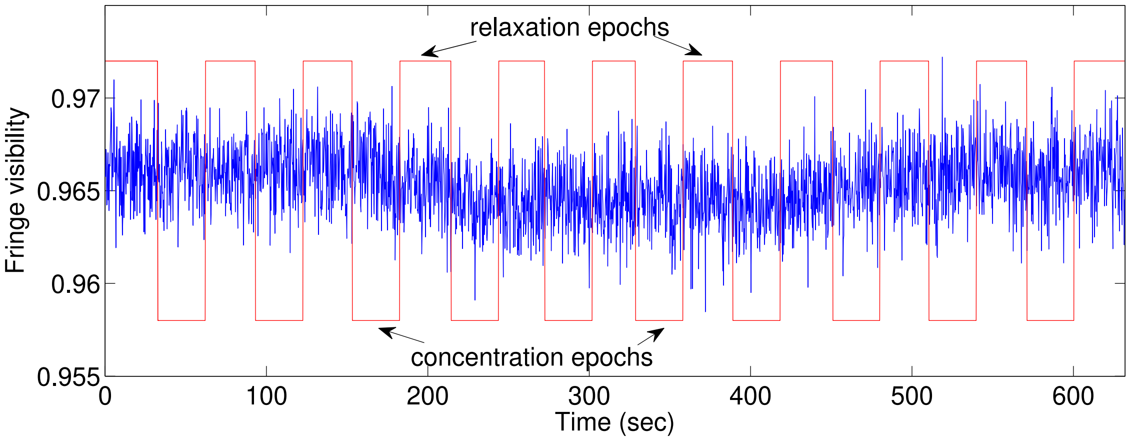

Fig 3. Fringe 9's visibility versus time for a typical session. The red square signal represents the concentration/relaxation epochs.

https://doi.org/10.1371/journal.pone.0211511.g003

Fig 3 shows fringe 9's visibility versus time during one typical session. The epochs, as sent by the server, are represented with the square signal: high values represent relaxation epochs, and low values concentration epochs.

For each session, we extract a single scalar value: the difference between the median of the fringe visibility during concentration epochs, and the median of the fringe visibility during relaxation epochs. The medians are considered as they are more robust to outliers than the average. Formally, given the fringe visibility time series fv, define fv^c (resp. fv^r) as the reduction of fv to the concentration (resp. relaxation) epochs, and Δv as the difference in median fringe visibility:

$$\Delta v = \text{median}(fv^c) - \text{median}(fv^r). \tag{2}$$

Δv is the statistics we will use in the following analyses.

2.3 Zero mean statistical testing

If the mind-matter interaction hypothesis is false, one would normally expect $\mathbb{E}(\Delta v)$ to be equal to zero. We test this by considering the values of Δv across all human sessions, and performing a zero-mean t-test. Note in passing that results are similar if one decides to estimate the variance of the values of Δv by bootstrapping before performing a z-test (given the large number of Δv values, this does not come as a surprise). It is common practice to remove outliers [20] before performing a mean test, given the sensitivity of the mean to outliers. We introduce q_{out} the percentage of outliers we discard from the list of Δv values. Given q_{out}, and a total number of session N, one removes the $q_{out}N/2$ highest and $q_{out}N/2$ lowest values of the list of Δv, before performing the test. In this first analysis, q_{out} is fixed to 20%. We will see later in Section 2.6 how this choice affects the results.

A time lag l is expected between the fringe visibility and the alternating instructions of concentration and relaxation. Indeed, a lag could occur for three main reasons: first due to the time one needs to switch one's attention from a concentration state to another, second due to the finite (and possibly slow) speed of the Internet connection, and third due to the 3 seconds span of the sliding window on which the feedback is computed. In the following, we will consider lags between 0 and 25 seconds.

The null hypothesis we are testing is therefore: H_0: *considering any time lag,* $\mathbb{E}(\Delta v)$ *is null.* Indeed, common sense suggests that whatever the concentration state of a participant, there is

no reason that the fringe visibility of the optical system should be affected. This hypothesis involves multiple testing ($m = 26$ tests precisely): one for each time lag l. For each time lag l we test the null hypothesis: H_0^l: *considering time lag* l, $\mathbb{E}(\Delta v)$ *is null*, that will output a t-value t_l and a p-value p_l. We then apply the Holm-Bonferonni method [21] to adjust for multiple comparison, and obtain an overall p-value p^{H_0} for H_0. To this end, sort p_l in ascending order to obtain p_l'. Consider p_l'' defined, for $k \in [1, 26]$, as $p_l''(k) = p_l'(k) * (m - k + 1)$. The overall p-value p^{H_0} is then formally defined as:

$$p^{H_0} = \min(p_l''). \tag{3}$$

This method is regarded as pessimistic in our context of correlated tests [22]. But in this controversial field of research, it is safer to use pessimistic estimations.

2.4 Preliminary results and remarks

Note that all p-values in this study are two-tailed. Fig 4 shows t_l and p_l versus the time lag l, for the human and control sessions of each year. The corrected p-value for multiple comparisons corresponding to H_0 for the human'13 sessions (resp. control'13, human'14, control'14) is $p^{H_0} = 4 \times 10^{-4}$ (resp. 0.3, 1, 1). These values call for a few preliminary observations. As in [1], we find that both years' control data act as expected by H_0 and that H_0 is rejected for the 2013's human sessions. More precisely, human sessions of 2013 show a significant anomaly towards negative values of Δv meaning that the median value of the fringe visibility is lower when the participant is concentrating than when the participant is relaxing. On the other hand, the observed shift towards positive values of Δv of the human sessions of 2014 is deemed insignificant. This does not match with the results of [1] where the authors find a significant shift towards positive t-values. This difference comes from the fact that we did not define the fringe visibility exactly as in [1]. We will explore in details in Section 2.8 the robustness of our results regarding the fringe visibility definition.

We now propose to make a very different choice in the analysis of this data than the one originally proposed. The authors in [1] propose to aggregate the data from both years, after inverting the sign of the 2014's Δv values to account for the feedback's sign inversion. We argue in this paper that aggregating the data is confusing and makes results' interpretation more difficult. It seems that aggregating the data after a sign inversion is an *ad-hoc* way of increasing the significance of the effect: there was indeed no reason to believe before the experiment that 2014's t-values would increase. In this preliminary analysis, 2014's data slightly shift towards high values, but within chance expectations. One could argue that aggregating the data after a sign inversion is using a possibly random fluctuation to one's advantage. Another

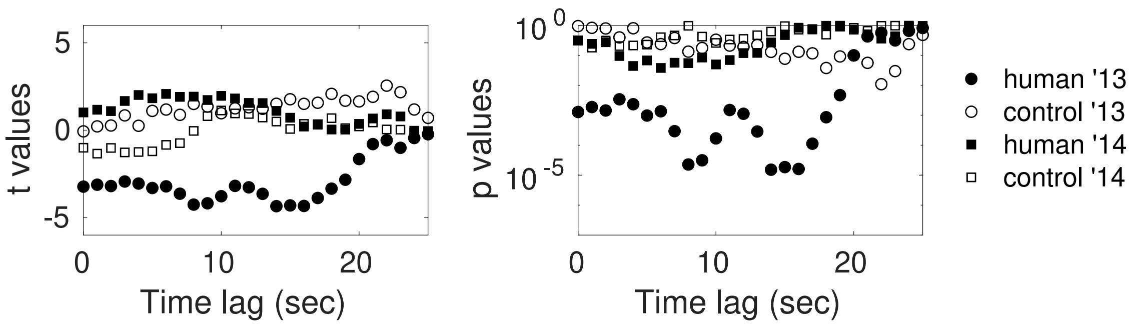

Fig 4. Result of zero-mean *t*-tests versus the time lag for fringe 9. t-values and p-values (corresponding to hypotheses H_0^l) versus the time lag, for the human and control sessions of each year; considering fringe 9, $q_{\text{out}} = 20\%$ and $\tau = 1000$.

https://doi.org/10.1371/journal.pone.0211511.g004

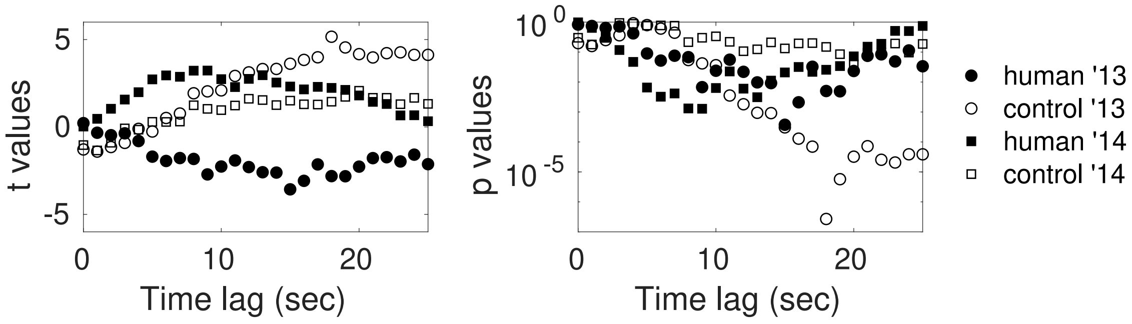

Fig 5. Result of zero-mean *t*-tests versus the time lag for fringe 7. *t*-values and *p*-values (corresponding to hypotheses H_l) versus the time lag, for the human and control sessions of each year; considering fringe 7, q_{out} = 20% and τ = 1000.

https://doi.org/10.1371/journal.pone.0211511.g005

possibility is to aggregate the data without the sign inversion. This is not reasonable given the fact that experimental conditions (specifically the feedback, which seems to be very important) were different for both years. The most reasonable decision regarding both years' analyses is to keep them separate—at the cost of lower statistical power.

Another fundamental difference between our analysis and the one proposed in [1] is prior knowledge regarding the time lag to consider. Authors in [1] build upon their previous (and independent) experiment [18] that indicated a time lag of 9 seconds as a good parameter to discriminate humans from controls (as long as the experiment used to learn this parameter and the experiment used to test this parameter are independent, this is perfectly possible). In our independent re-analysis, we prefer the safer choice of no prior knowledge, thereby necessarily testing all time lags followed by constraining adjustments due to multiple testing—at the cost, once again, of lower statistical power.

In the next four sections (Section 2.5 to Section 2.8), we look at the robustness of the results regarding all the seemingly arbitrary decisions we made at every step of this pre-analysis, namely: the fringe number to consider (we chose fringe 9), the outlier percentage q_{out} (we chose q_{out} = 20%), the length threshold τ under which we deem sessions too short to give any reasonable estimation of Δv (we chose τ = 1000 camera frames), and *fv*'s estimation method (we chose the normalized difference between spline interpolated envelopes). In addition, in Section 2.9, we test the robustness of the results with respect to random subsampling of the data.

2.5 Extending the analysis to all fringes

From our re-analysis point-of-view, fringe number 9 is an arbitrary choice (fringe number 9 was originally chosen in [1] as a good parameter choice to discriminate humans from controls in their previous independent experiment [18]) and it is necessary to look at other fringes. Fig 5 shows results obtained for fringe number 7: the human sessions' anomalies are in the same direction than for fringe number 9, with a less (resp. more) significant result for 2013 (resp. 2014) with a corrected p-value of $p^{H_0} = 1 \times 10^{-2}$ (resp. 3×10^{-2}). The big surprise comes from the 2013 control sessions that show a significant ($p^{H_0} = 7 \times 10^{-6}$) increase of Δv. Once again, this is different than the results shown in Fig 2 of [1] where the 2013 controls are within chance expectation for all fringes. This is mainly due to the combination of two facts: i/ they suppose a prior knowledge of a 9 second time lag and we do not, and ii/ large anomalies of the 2013 control data occur after 9 seconds—see Fig 5.

To look at all fringes at once, Fig 6 shows the corrected p-values p^{H_0} as a function of the fringe number for all four different session types. We see how a particular choice of fringe for

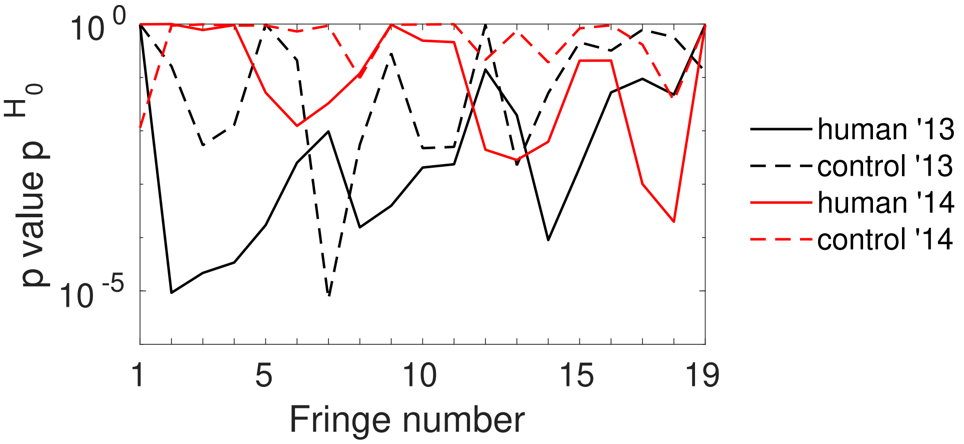

Fig 6. Corrected for multiple comparisons *p*-values corresponding to hypothesis H_0 for the human and control sessions of each year as a function of the fringe number; considering q_{out} = 20% and τ = 1000.

https://doi.org/10.1371/journal.pone.0211511.g006

the analysis is problematic: depending on this choice one may serve different outcomes of the statistical test! For instance, one could *p*-hack and choose *a posteriori* fringe number 5 as a good candidate to discriminate humans from controls; or choose fringe number 19 to conclude that one cannot discriminate one from the other.

To go further, and in order to prevent us from choosing the fringe number(s) that serve one hypothesis or the other, we propose two strategies that both take into account information from all fringes.

A new null hypothesis. We propose to investigate a new null hypothesis comprehending all fringes: H_0': *considering any time lag and any fringe number*, $\mathbb{E}(\Delta v)$ *is null*. Testing H_0' implies doing m' = 26 * 19 = 494 individual tests (26 time lags for each of the 19 fringes). We correct for multiple comparisons using the same Holm-Bonferonni method that becomes even more conservative given that we add many correlated tests. Keeping that in mind, we obtain a corrected *p*-value for the 2013 human (resp. 2013 control, 2014 human, 2014 control) sessions of $p^{H_0'} = 2 \times 10^{-4}$ (resp. 10^{-4}, 4×10^{-3}, 0.2). Fig 7 shows the *t*-value t_l of each of the 494 individual tests versus the time lag and the fringe number: the direction from which the data differs from the null hypothesis H_0^l is consistent across all individual tests. Both human sessions differ significantly from H_0 with an anomaly towards negative (resp. positive) t-values for 2013 (resp. 2014). The control sessions of 2013 differ significantly from H_0 with an anomaly towards positive *t*-values while the 2014 controls are within chance expectation.

A new fringe visibility definition. The variability observed in Fig 6 could be due to a signal-to-noise ratio (SNR) that is too small for our task. In order to increase the SNR, we define $\overline{fv}_\mu$ the average of *fv* over all fringes between 10 − μ and 10 + μ (with μ an integer between 0 and 9). We choose to concentrate on intervals centered around fringe 10 as it is the one with the best SNR. We could of course choose other intervals to average over but we would encounter the very same problem we are trying to avoid: different intervals will serve different hypotheses and a particular choice of interval would be difficult to justify. Here, we rely on the (strong) SNR argument to choose to look at all intervals centered around fringe 10.

Given this new definition of fringe visibility, we test the null hypothesis: H_0'': *considering any time lag and any* μ, $\mathbb{E}(\Delta v)$ *is null*. Testing H_0'' implies doing m'' = 26 * 10 = 260 individual tests (26 time lags for each of the 10 possible choices for μ). After correction for multiple comparisons, we obtain a corrected *p*-value for the 2013 human (resp. 2013 control, 2014 human, 2014

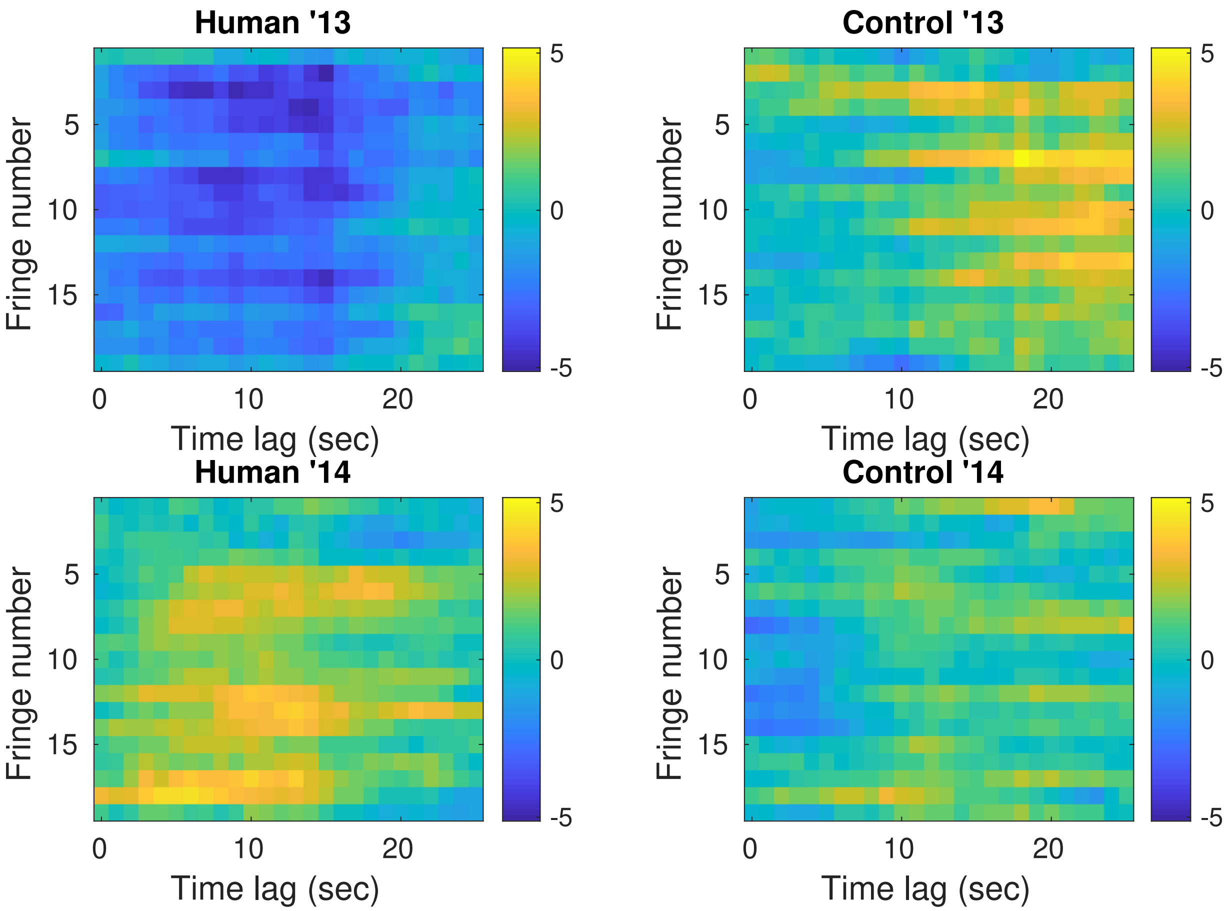

Fig 7. *t*-values of all tests performed in H_0'. *t*-values t_l of each of the 494 individual tests versus the time lag and the fringe number for all four different types of sessions; considering $q_{out} = 20\%$ and $\tau = 1000$.

https://doi.org/10.1371/journal.pone.0211511.g007

control) sessions of $p^{H_0''} = 4 \times 10^{-4}$ (resp. 2×10^{-2}, 0.2, 1). Fig 8 shows the *t*-value of each of the 260 individual tests versus the time lag and μ: the direction from which the data differs from the null hypothesis is the same than previously.

Summary. We first observed that results are not robust with respect to the choice of fringe number one studies. To avoid choosing a fringe number, we i/ performed a test whose null hypothesis encompasses all fringe numbers, ii/ performed a test on the average of the fringe visibility over central fringes. Both analyses show the following:

- the 2013 human sessions shift significantly towards negative $\Delta\nu$ values.
- the 2013 control sessions shift significantly towards positive $\Delta\nu$ values.
- the 2014 control sessions are insignificant.
- In both analyses, we observe a shift of the 2014 human sessions towards positive $\Delta\nu$ values. Nevertheless, testing against H_0' deems this shift significant, whereas testing against H_0'' does not.

In the following robustness investigations, and given the difficulty to choose between H_0' and H_0'', we will systematically consider both.

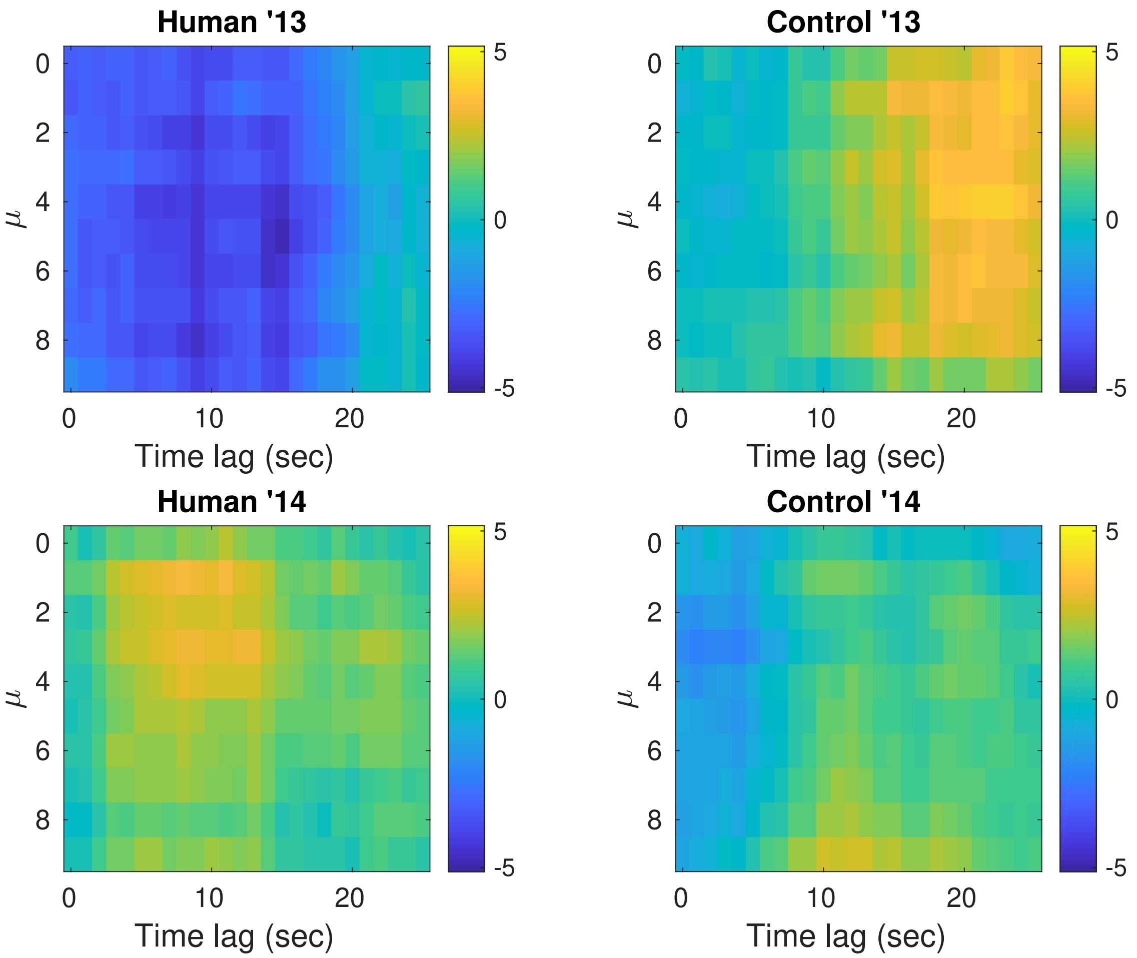

Fig 8. *t*-values of all tests performed in H_0”. *t*-values t_l of each of the 260 individual tests versus the time lag and μ for all four different types of sessions; considering $q_{out} = 20\%$ and $\tau = 1000$.

https://doi.org/10.1371/journal.pone.0211511.g008

2.6 Robustness regarding the outlier percentage q_{out}

Fig 9 shows the *p*-values $p^{H_0'}$ and $p^{H_0''}$ for the four different types of sessions versus the outlier percentage q_{out}. As expected when trimming outliers of truly anomalous data, the significance of the effects increase when q_{out} increases. The direction of the significant shifts (not shown)

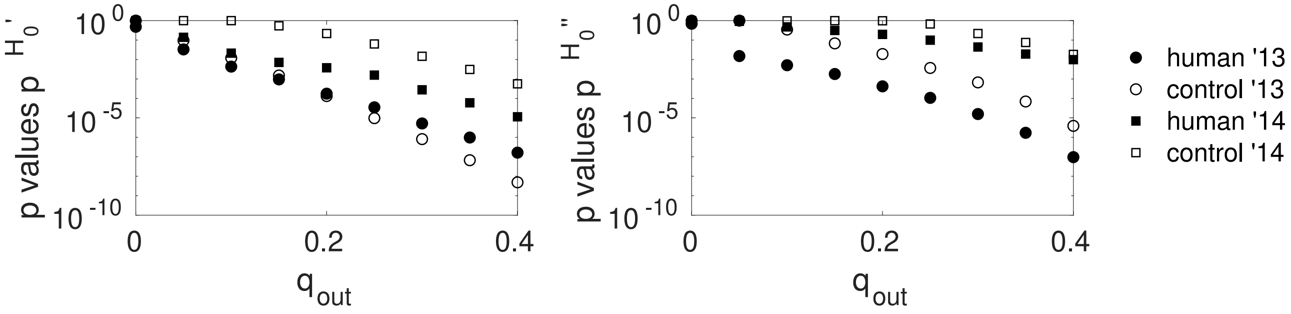

Fig 9. Robustness regarding the outlier percentage q_{out}. Corrected for multiple comparisons *p*-values corresponding to hypotheses H_0' (left) and H_0'' (right) for the four types of sessions as a function of the outlier percentage q_{out}; considering $\tau = 1000$.

https://doi.org/10.1371/journal.pone.0211511.g009

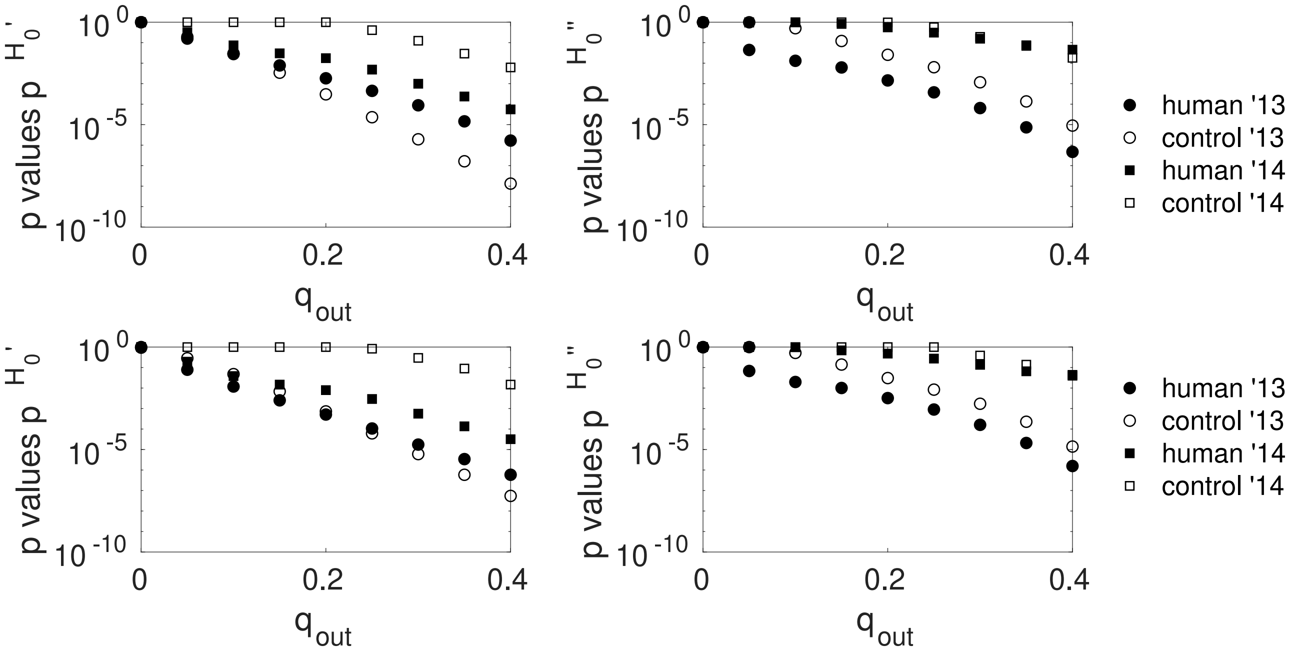

Fig 10. Robustness regarding the session length threshold τ. Corrected for multiple comparisons p-values corresponding to hypotheses H_0' (left) and H_0'' (right) for the four types of sessions as a function of the outlier percentage q_{out}, for length thresholds $\tau = 1700$ (top) and $\tau = 2300$ (bottom).

https://doi.org/10.1371/journal.pone.0211511.g010

do not change and are as previously stated. The slightly significant results that arise in the 2014 control data for high outlier percentage when testing hypothesis H_0' comes from a negative shift at small time lags (of which we see some indication in the bottom right figure of Fig 7). Apart from this, results as summarized at the end of Section 2.5 are robust with respect to q_{out}.

2.7 Robustness regarding the length threshold τ

We recall that τ is the threshold under which we deem sessions too short to estimate Δv correctly. Fig 10 shows the p-values $p^{H_0'}$ and $p^{H_0''}$ versus q_{out} for two other values of τ: the results are robust regarding the length threshold. In the following, we consider only results obtained with $\tau = 1000$.

2.8 Robustness regarding the fringe visibility estimation method

Until now we have been using the normalized difference between the interpolated envelopes as the definition of the fringe visibility (see Eq (1)). It is necessary to look at the sensitivity of the results with regards to that method of estimation. Authors in [1] define the visibility of fringe n as the normalized difference between the n-th local maximum M_n and its preceding local minimum m_n:

$$fv = \frac{M_n - m_n}{M_n + m_n}. \quad (4)$$

Results obtained with this definition on fringe 9, and with $q_{out} = 20\%$ and $\tau = 1000$, are shown in Fig 11 (top). As in [1], we observe significant anomalies in the human data of both years especially around $l = 9$ seconds, and insignificant results for the controls. Fig 11 (middle) gives the bigger (and corrected for multiple comparisons of the time lag) picture by plotting the p-value p^{H_0} for the four types of sessions versus the fringe number. This figure may be directly

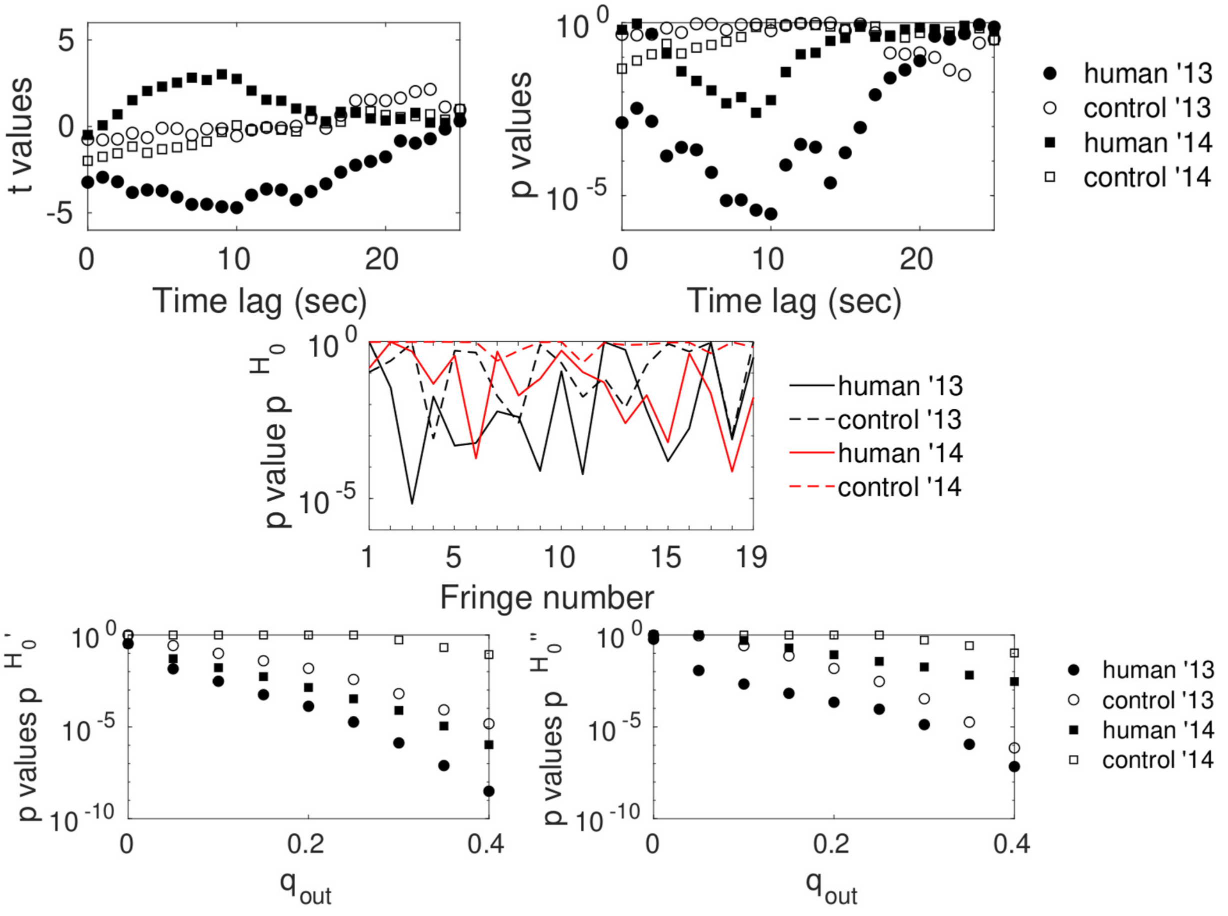

Fig 11. Test results using Eq 4 to define the fringe visibility. (top) *t*-values t_l and *p*-values p_l versus the time lag for fringe number 9 (with q_{out} = 20%), (middle) *p*-value p^{H_0} versus the fringe number (with q_{out} = 20%) and (bottom) *p*-value $p^{H_0'}$ (left) and $p^{H_0''}$ (right) versus the outlier percentage.

https://doi.org/10.1371/journal.pone.0211511.g011

compared to Fig 2 of [1] (modulo the fact that we plot *p*-values and they plot bootstrapping *z*-scores): we observe a similar behavior for the human data results but significant anomalies in the 2013 controls that are not visible in [1]. This is mainly due to the prior knowledge they have on the time lag that we do not suppose.

Once again, depending on the fringe one considers, one may be lead to contradictory conclusions. One therefore needs to consider hypotheses H_0' and H_0''. Fig 11 (bottom) shows the *p*-values $p^{H_0'}$ and $p^{H_0''}$ versus the outlier percentage q_{out}.

For a fringe number n and its associated local maximum M_n, there is no reason to define its visibility by comparing M_n to its previous local minimum m_n rather than its succeeding local minimum m_{n+1}. If one defines

$$fv = \frac{M_n - m_{n+1}}{M_n + m_{n+1}}, \tag{5}$$

then one obtains the overall results of Fig 12.

Comparing Figs 9, 11 (bottom) and 12, as well as looking at the associated *t*-values (not shown), one concludes that the results as summarized at the end of Section 2.5 are robust with respect to the fringe visibility estimation method.

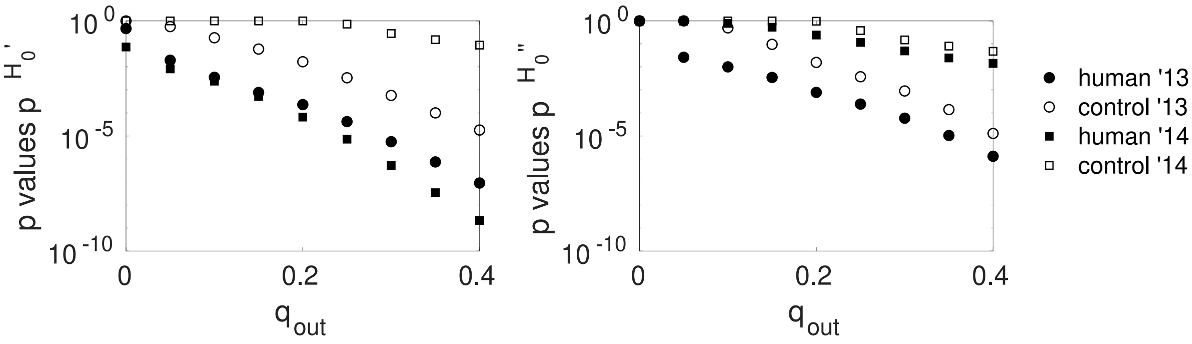

Fig 12. Test results using Eq 5 to define the fringe visibility. p-values $p^{H_0'}$ (left) and $p^{H_0''}$ (right) for the four types of sessions versus the outlier percentage q_{out}.

https://doi.org/10.1371/journal.pone.0211511.g012

2.9 Robustness after random subsampling of sessions

In order to probe whether the observed anomalies are not due to a random fluctuation pertaining to this particular set of sessions, we explore the robustness of the observed p and t-values with respect to random subsampling of the data. For each four types of sessions (human and control of both years), we subsample uniformly without replacement 75% of the data before testing H_0' and H_0'' (with the fringe visibility method of Eq (1), q_{out} = 20% and τ = 1000). We perform this 1000 times and plot in Fig 13 the histograms of $p^{H_0'}$, $p^{H_0''}$, as well as the histograms

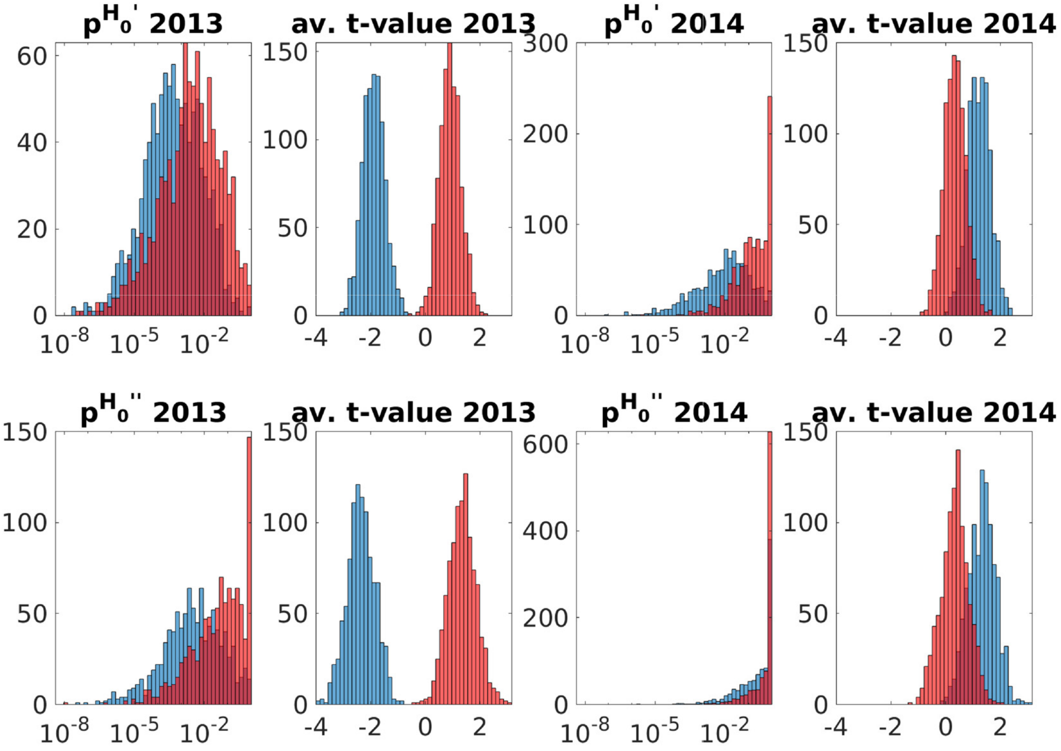

Fig 13. Robustness to random subsampling of the data. (top) Histograms of $p^{H_0'}$ and its associated average t-value for both years. (bottom) Histograms of $p^{H_0''}$ and its associated average t-value for both years. Blue histograms represent human data. Red histograms represent control data. The histograms are plotted for 1000 random subsamples of the data (randomly keeping 75% of each four categories), with the fringe visibility method of Eq (1), q_{out} = 20% and τ = 1000.

https://doi.org/10.1371/journal.pone.0211511.g013

of their associated average t-value. The histograms of human p-values are systematically shifted towards smaller values compared to their control counterpart. The bulk of the 2014 control p-values is concentrated around 1 for both hypotheses and the p-values of the 2014 human sessions are either around 0.01 for H_0' or concentrated around 1 for H_0''. The p-value histograms for the 2013 data are also consistent with what we have previously observed. Looking at the average t-values, we clearly observe a shift towards negative values for the 2013 human data and a shift towards positive values for the 2013 control and the 2014 human data. There is no clear shift for the 2014 controls. Thus, the results as summarized at the end of Section 2.5 are robust to random subsampling of the data.

It follows from all these analyses that anomalies exist in the data beyond any reasonable doubt: whichever pre-analysis parameters or methods one chooses, one still observes anomalies, even considering a conservative multiple comparison adjustment. The question now arises as to whether this is due to artifacts inherent to the experimental process. We address this in the following section.

2.10 Time-matched sessions

The left (resp. right) figure of Fig 14 shows the number of recorded control (resp. human) sessions per day during the two years of the experiment. The control sessions show important gaps in the recording, and a potential bias could arise because some human sessions were recorded during a period of the year (or of the day) with very different temperature or humidity conditions than control sessions.

To prevent this, we consider time-matched data: we only keep pairs of human and control sessions that were recorded within one hour of each other; and discard all isolated sessions. This brings down the data to 326 pairs of sessions in 2013 and 647 pairs of sessions in 2014. Fig 15 shows the p-values $p^{H_0'}$ and $p^{H_0''}$ versus q_{out}. Comparing this figure to Fig 9, one observes that the 2013 controls are now deemed insignificant by both tests, whereas the 2014 controls are now deemed significantly anomalous! Even more surprising: this result is robust with respect to the minimal session length τ and to the fringe visibility estimation method (not shown). Note also that the directions of the shifts in Δv are still the same as previously stated. To probe if this result is only a random fluctuation due to this specific subsampling, we may compare it to results obtained on randomly chosen subsamples of same size (that is: 326 for the 2013 data, and 647 for the 2014 data). To this end, we plot in S1 Fig the histograms (obtained on a 1000 random such subsamples, in the case $q_{\text{out}} = 20\%$ and $\tau = 1000$) of p-values and average t-values. These histograms suggest that the obtained p-values on time-matched sessions are not extraordinary for subsamples of such size. The sudden significance of the 2014 control data could simply be a random fluctuation due to subsampling. The big difference

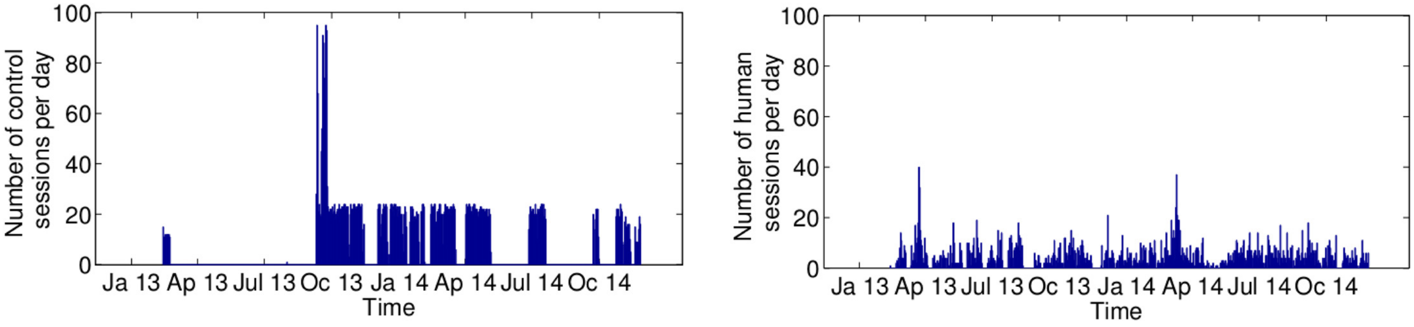

Fig 14. Number of sessions recorded per day. Number of sessions recorded per day during 2013 and 2014 for the control (left) and the human (right) sessions.

https://doi.org/10.1371/journal.pone.0211511.g014

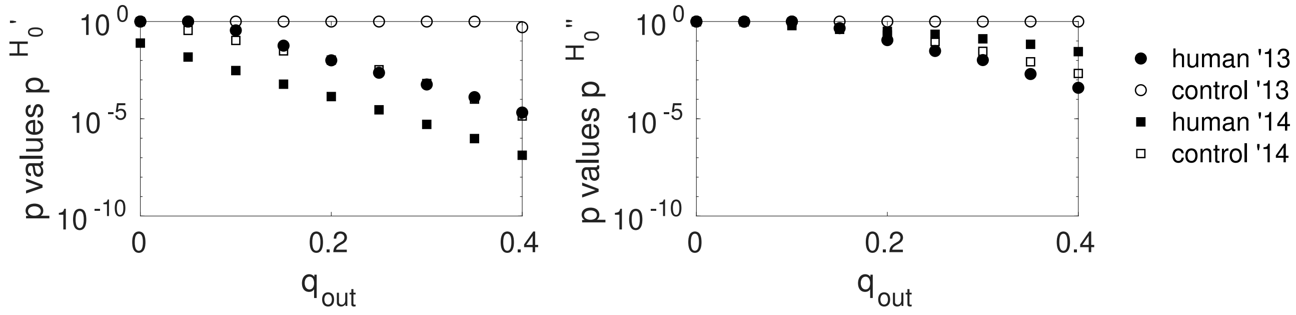

Fig 15. Test results for time-matched data. Results for time-matched data for $\tau = 1000$ and using the spline interpolation estimation method of Eq (1) for the fringe visibility. Left: $p^{H_0'}$ versus q_{out}. Right: $p^{H_0''}$ versus q_{out}.

https://doi.org/10.1371/journal.pone.0211511.g015

between Fig 13 and S1 Fig is a general shift of all p-values towards larger values, which is expected when decreasing the number of sessions to test. Importantly, the histograms of human data are still slightly shifted towards lower p-values compared to their control counterpart, indicating that anomalies still persist.

We perform a last experiment on this time-matched data in our pursuit of a possible systematic bias. Instead of testing if both the human and the control data have zero mean, we test whether the human and control data have the same mean (whatever it may be) in order to see if we can detect any potential experimental drifts accounting for the non-null averages. To this end, and similarly to previously, we define hypothesis G_0': *Given any time lag and any fringe number*, $\mathbb{E}(\Delta v)$ *is the same for time-matched human and control data*; as well as hypothesis G_0'': *Given any time lag and any* μ, $\mathbb{E}(\Delta v)$ *is the same for time-matched human and control data*. We test these two hypotheses using a standard two-sample t-test, for different values of q_{out} and plot the p-values and the average t-values over the individual tests in Fig 16. We observe a decrease of the anomalies' significance (note the change of scales in the p-values figures compared to Fig 15) but they do not disappear. Once again, we observe that the average of Δv for the 2013 human sessions is significantly lower than the average of Δv for its time-matched control counterpart; whereas the average of Δv for the 2014 human sessions is significantly larger than the average of Δv for its time-matched control counterpart. This decrease in significance may indicate that there is a systematic bias (an experimental drift for instance) that makes the average of Δv drift systematically away from 0 (one would nevertheless still need to account for drifts going in opposite directions for human and control data). In S2 Fig, we plot the histograms of the p and t values of the same-mean t-test obtained in the same subsampling condition as for S1 Fig. A strong indication that we are facing a systematic bias would be to record very large p-values (that is: insignificant tests) compared to the bulk obtained in the histograms, which is not the case here. Thus, we observe that anomalies do persist even if looking at the same-mean test of time-matched data. Note that we repeated the same experiment for closer time-matched sessions separated by only 15 and 30 minutes (not shown) and made similar observations.

2.11 Effect sizes

p-values are not the only statistical variable one should pay attention to: effect sizes are equally important to control and help estimate how strong is an observed anomaly. For each test l (out of the 494 for H_0' and the 260 for H_0''), we estimate its effect size e_l with:

$$e_l = \frac{t_l}{\sqrt{N_l}}, \tag{6}$$

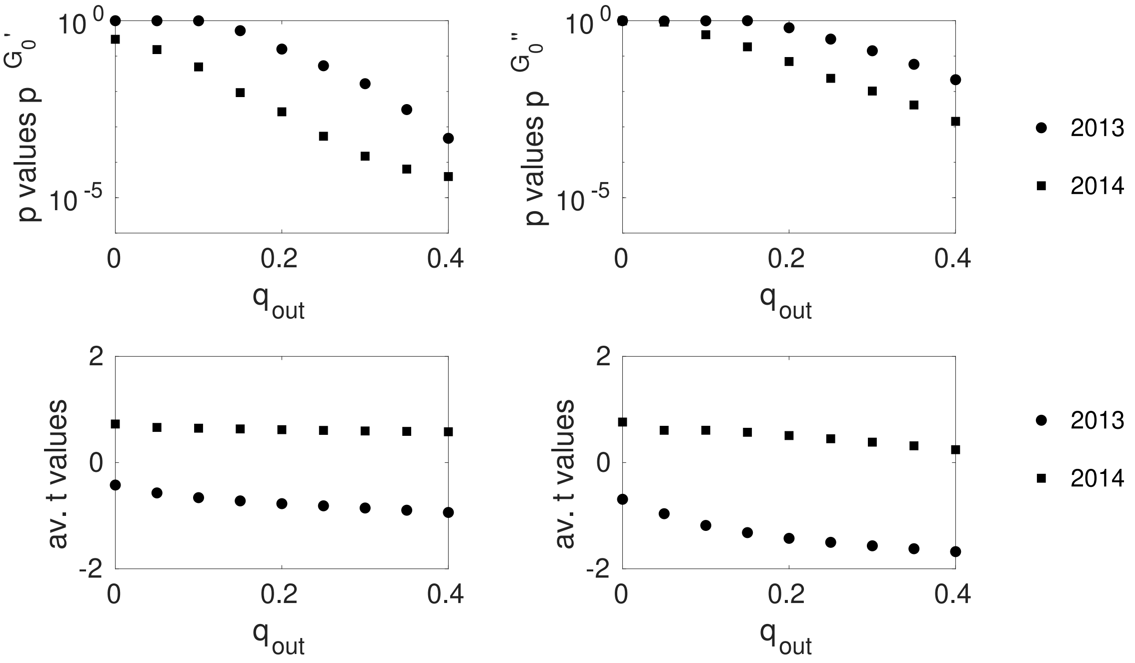

Fig 16. Same-mean test results for time-matched data. Results of testing whether the statistics $\Delta\nu$ of time-matched human and control data have the same mean. (Left): testing hypothesis G_0'. (Right): testing hypothesis G_0''. The bottom figures show the average over all individual tests of G_0' (left) and G_0'' (right) of the t-values. A positive (resp. negative) t-value means that human data have a lower (resp. higher) mean than control data. Results are obtained with $\tau = 1000$ and using the spline interpolation estimation method of Eq (1) for the fringe visibility.

https://doi.org/10.1371/journal.pone.0211511.g016

where N_l is the number of sessions involved in the test. We report in Table 1 the maximum and mean of the absolute effect size of each of all the individual tests for both H_0' and H_0'', considering either all sessions, or only time-matched sessions. The effect sizes are small, but not negligible. Interestingly, the average effect sizes are systematically slightly higher for the human sessions than for the controls.

3 Discussion

The preliminary analysis proposed in Section 2.4 is subject to four seemingly arbitrary choices: the fringe number, the minimal length of a session, the outlier percentage and the choice of

Table 1. Effect sizes.

H_0'	all data				time-matched data			
session type	H'13	C'13	H'14	C'14	H'13	C'13	H'14	C'14
max($\|e_l\|$)	0.16	0.12	0.12	0.07	0.27	0.16	0.23	0.19
mean($\|e_l\|$)	0.07	0.03	0.04	0.02	0.08	0.04	0.08	0.07
H_0''	all data				time-matched data			
session type	H'13	C'13	H'14	C'14	H'13	C'13	H'14	C'14
max($\|e_l\|$)	0.15	0.09	0.09	0.05	0.22	0.12	0.14	0.15
mean($\|e_l\|$)	0.09	0.04	0.04	0.02	0.11	0.05	0.08	0.08

Maximum and mean of the absolute effect size of each of all the individual tests against H_0^l, either considering all the data, or only the time-matched data; with q_{out} = 20%, $\tau = 1000$, and the spline interpolation estimation method for the fringe visibility. "H" stands for Humans, and "C" for Controls.

https://doi.org/10.1371/journal.pone.0211511.t001

the fringe visibility estimation method. In Section 2.5, we observe that different fringe choices change the output of the statistical tests, and thus the conclusions that may be drawn from the data. We therefore propose two more robust methods that avoid choosing fringes: the first one is to encompass all fringes in the null hypothesis, leading to H_0', and the second is to average the fringe visibility over central intervals of fringes, leading to H_0''. We summarize the observed results of the tests at the end of Section 2.5. We not only show that these results are robust i/ regarding the minimal session length in Section 2.7, ii/ regarding the fringe visibility estimation method in Section 2.8, iii/ to random subsampling of the data in Section 2.9; but we also show in Section 2.6 that the significance of the anomalies increase as the percentage of outliers increases, which is a strong indication that the core of the data is truly anomalous.

Given the conservative nature of the Holm-Bonferonni correction for multiple comparison we used to test H_0 and ultimately H_0' and H_0'', the results obtained here show that, however one chooses to study the data, it is undeniable that:

- the human data of 2013 shows anomalies towards negative values of $\Delta\nu$.
- the control data of 2013 shows anomalies towards positive values of $\Delta\nu$.

The 2014 data is not as clear but one still observes that:

- the human data of 2014 is, to simplify, deemed anomalous by H_0' and not by H_0''. When deemed anomalous, the shift of $\Delta\nu$ is observed towards positive values.
- the control data of 2014 is deemed insignificant in the vast majority of our experiments.

Disturbing as it is to find anomalous behavior in the 2013 controls, it should not overshadow the fact that this paper is the third independent statistical analysis (after [1, 19]) showing significant differences in fringe visibility between concentration and relaxation epochs of human subjects. Moreover, it is shown that the system responds in the same direction as what is asked to the human participants: in 2013, the median value of the fringe visibility is statistically lower when the participant is concentrating than when the participant is relaxing; in 2014, the contrary is observed (even though with less significance). The working hypothesis presented in the introduction states that human intention could be considered as a weak measurement of the system, thereby slightly collapsing its wave function. The 2013 human dataset is in favor of this hypothesis; whereas the 2014 human dataset is not: the 2014 detected anomaly is even in the opposite direction than what is predicted by the hypothesis. Hence, the data does not seem to be consistent with a gain in which-way information and other working hypotheses, such as Pradhan's [13] already mentioned in the introduction, should be considered. Note that the only difference between both years' datasets is the sign of the feedback, thereby suggesting to work on new hypotheses in future replication attempts that should specifically take into account the feedback. Note also that the human sessions are in a sense already control-proofed by nature: they compare relaxation epochs (that could be considered as controls) to concentration epochs.

Nevertheless, unlike the authors of [1], we argue that this result does not hold as a strong evidence of mind-matter interaction as long as the 2013 control sessions show almost equally significant effects. Two types of arguments could account for significant controls. The first one involves some sort of hysteresis of the system. Indeed, if one considers the mind matter interaction hypothesis as a working hypothesis, then one can also imagine that after a human session, the system keeps, at least for some time, some memory of its interaction with a human subject; giving rise to significant controls. If that was the case, one would expect the significance of the controls to fade away with respect to the time gap between the control's time of measure and its previous human session. Further analysis of the data shows no indication

supporting this hypothesis. The second type of arguments involves a systematic bias in the experiment's protocol. This is of course the most reasonable type of arguments, but one still needs to explain what specific bias could explain the following points.

- Significant results imply a 30 seconds periodicity in the fringe visibility data, as the overall Δv measure of a given session is the average over 21 alternating epochs of 30 seconds. Where does this period come from?
- The direction of the anomalies is very robust and varied: the 2013 human (resp. control) sessions show anomalies towards negative (resp. positive) Δv; the 2014 human sessions show anomalies towards positive Δv. What bias could explain such robust direction of anomalies?

In an attempt to control possible biases due to difference in temperature or humidity conditions or some other sort of experimental drift, we discuss in Section 2.10 results obtained on time-matched data. We perform zero-mean t-tests for time-matched sessions in both years, as well as same-mean t-tests. We observe a decrease in the significance of the anomalies but not more than what could be expected when decreasing the number of sessions to test. We did not find strong evidence of a systematic bias explaining the observed anomalies. In future replication attempts, and in order to precisely account for any experimental drift, an idea would be to split the beam at the exit of the laser, in order to make it interfere through two (nearby) double-slits: one for the experiment, and the other to monitor simultaneously all fluctuations due for instance to temperature, pressure, or laser intensity variations.

Before we conclude, let us make an important statement. We have made many statistical tests, and to prevent p-hacking, one needs to look at all these tests as a whole. Extracting one test or the other from the whole is not recommended. Note that, on top of the tests discussed in the paper we have also performed tests with two other fringe visibility definitions: the average of Eqs (4) and (5), and the fringe visibility extracted by spline interpolation as in Eq (1) but sampled only at the extrema instead of considering the average over each fringe as presented here. None of these tests showed a significant difference than the ones shown in the paper.

4 Conclusion

The thorough analysis pursued in this paper gives a much broader and full picture of the data than the ones previously published in [1] and [19]. On the one hand, we find undeniable anomalies in the human data with shifts of the fringe visibility in the direction expected by human intention. The fact that fringe visibility decreases when human intention tries to make it decrease, and increases when human intention tries to make it increase is remarkable. On the other hand, significant anomalies are found in the 2013 controls with shifts in the opposite direction than the anomalies in the human data. Effect sizes show a small (none exceeding 0.11) but non-negligeable size of the effect, with human effect sizes slightly higher than control ones. Our efforts to find systematic biases explaining these anomalous shifts are not conclusive. Finally, all our analysis and figure-plotting Matlab codes, along with all the raw data, are publicly available on the Open Science Framework database platform at the address https://osf.io/ywktp/, to aid further investigations on this particular dataset.

As far as data analysis is concerned, this dataset does not allow us to make any further conclusions. Interpretation of these results is challenging. Given the behaviour of the controls, we argue that these results cannot be fully interpreted as evidence of mind-matter interaction. Even with well-behaved controls, multiple replications would be needed. The fact that the results reported here remain inexplicable should not undermine the significance of the detected anomalies. Exploration of the quantum measurement problem with an open-minded yet rigourously scientific point of view, is an important endeavour, and has a major potential

impact on our current understanding of reality. Given the controversial aspect of this research, attempts to reproduce such an experiment should be done by groups of experts from different fields of research including quantum mechanics, neuroscience and statistics, both skeptics and believers, collaborating to design the most rigourous protocol. Personal beliefs, may they be strongly in favor or against the mind-matter interaction hypothesis, have to be put aside, to collectively pursue a clear and objective investigation of this particular interpretation of the quantum measurement problem.

Supporting information

S1 Fig. Robustness to random subsampling. (top) Histograms of $p^{H'_0}$ and its associated average *t*-value for both years. (bottom) Histograms of $p^{H''_0}$ and its associated average *t*-value for both years. Blue histograms represent human data. Red histograms represent control data. The histograms are plotted for 1000 random subsamples of the data (randomly keeping 326 sessions of each category in the 2013 data and 647 sessions of each category in the 2014 data), with the fringe visibility method of Eq (1), $q_{\text{out}} = 20\%$ and $\tau = 1000$.
(EPS)

S2 Fig. Robustness to random subsampling of the same-mean test. (top) Histograms of $p^{G'_0}$ and its associated average *t*-value for both years. (bottom) Histograms of $p^{G''_0}$ and its associated average *t*-value for both years. A positive (resp. negative) *t*-value means that human data have a lower (resp. higher) mean than control data. The histograms are plotted for 1000 random subsamples of the data (randomly keeping 326 sessions of each category in the 2013 data and 647 sessions of each category in the 2014 data). Results obtained with the fringe visibility method of Eq (1), $q_{\text{out}} = 20\%$ and $\tau = 1000$.
(EPS)

Acknowledgments

We would like to thank the authors of [1] for an unlimited access to both years' data, and their patience in answering all our questions regarding technical details of the data and the experimental protocol. Also, we would like to thank the anonymous reviewers for their insightful comments that greatly improved the quality of this manuscript.

Author Contributions

Conceptualization: Nicolas Tremblay.

Data curation: Nicolas Tremblay.

Formal analysis: Nicolas Tremblay.

Funding acquisition: Nicolas Tremblay.

Investigation: Nicolas Tremblay.

Methodology: Nicolas Tremblay.

Project administration: Nicolas Tremblay.

Resources: Nicolas Tremblay.

Software: Nicolas Tremblay.

Supervision: Nicolas Tremblay.

Validation: Nicolas Tremblay.

Visualization: Nicolas Tremblay.

Writing – original draft: Nicolas Tremblay.

Writing – review & editing: Nicolas Tremblay.

References

1. Radin D., Michel L. and Delorme A. Psychophysical modulation of fringe visibility in a distant double-slit optical system. *Physics Essays*, vol. 29, number 1, pp 14–22, 2016. https://doi.org/10.4006/0836-1398-29.1.014
2. Von J.Neumann, Mathematical foundations of quantum mechanics. Princeton university press. 1955.
3. Wigner E. and Margenau H., Symmetries and reflections, scientific essays. *American Journal of Physics*, vol 35, number 12, pp 1169–1170, 1967. https://doi.org/10.1119/1.1973829
4. Stapp H., Quantum theory and the role of mind in nature. *Foundations of Physics*, vol 31, number 10, pp. 1465–1499, 2001. https://doi.org/10.1023/A:1012682413597
5. Schlosshauer M., Kofler J. and Zeilinger A., A snapshot of foundational attitudes toward quantum mechanics. *Studies in History and Philosophy of Science Part B: Studies in History and Philosophy of Modern Physics*, vol 44, number 3, pp 222–230, 2013. https://doi.org/10.1016/j.shpsb.2013.04.004
6. Jammer M., The Philosophy of Quantum Mechanics: The Interpretations of Quantum Mechanics in Historical Perspectives. John Wiley, 1974.
7. Schlosshauer M., Decoherence, the measurement problem, and interpretations of quantum mechanics. *Reviews of Modern Physics*, vol 76, number 4, 2005. https://doi.org/10.1103/RevModPhys.76.1267
8. Everett H., "Relative state" formulation of quantum mechanics. *Reviews of modern physics*, vol 29, number 3, 1957. https://doi.org/10.1103/RevModPhys.29.454
9. Ibison M., and Jeffers S., A double-slit diffraction experiment to investigate claims of consciousness-related anomalies. *Journal of Scientific Exploration*, vol 12, pp. 543–550, 1998.
10. Feynman R., The Feynman Lectures on Physics. Volume III: Quantum Mechanics. Addison-Wesley, 1966.
11. Englert B., Fringe visibility and which-way information: An inequality. *Physical review letters*, vol 77, number 11, 1996. https://doi.org/10.1103/PhysRevLett.77.2154
12. Dürr S., Nonn T. and Rempe G., Fringe visibility and which-way information in an atom interferometer. *Physical review letters*, vol 81, number 26, 1998.
13. Pradhan R., An explanation of psychophysical interactions in the quantum double-slit experiment. *Physics Essays*, vol. 28, number 3, pp 324–330, 2015. https://doi.org/10.4006/0836-1398-28.3.324
14. Pradhan R., Psychophysical Interpretation of Quantum Theory. *NeuroQuantology*, vol. 10, number 4, pp 629–646, 2012. https://doi.org/10.14704/nq.2012.10.4.592
15. Sassoli de Bianchi M., Quantum measurements are physical processes. Comment on" Consciousness and the double-slit interference pattern: Six experiments," by Dean Radin et al.[Phys. Essays 25, 157 (2012)]. *Physics Essays*, vol. 26, number 1, pp 15–20, 2013.
16. F. Pallikari, On the question of wavefunction collapse in a double-slit diffraction experiment. *arXiv*, 1210.0432, 2012.
17. Radin D., Michel L., Galdamez K., Wendland P., Rickenbach R. and Delorme A., Consciousness and the double-slit interference pattern: Six experiments. *Physics Essays*, vol. 25, number 2, pp 157–171, 2012. https://doi.org/10.4006/0836-1398-25.2.157
18. Radin D., Michel L., Johnston J. and Delorme A., Psychophysical interactions with a double-slit interference pattern. *Physics Essays*, vol. 26, number 4, pp 553–566, 2013. https://doi.org/10.4006/0836-1398-26.4.553
19. Baer W., Independent verification of psychophysical interactions with a double-slit interference pattern. *Physics Essays*, vol 8, number 1, pp. 47–54, 2015. https://doi.org/10.4006/0836-1398-28.1.47
20. Osborne J. and Overbay A., The power of outliers (and why researchers should always check for them). *Practical assessment, research & evaluation*, vol 9, number 6, 2004.
21. Holm S., A simple sequentially rejective multiple test procedure. *Scandinavian journal of statistics*, pp. 65–70, 1979.
22. Abdi H., Holm's sequential Bonferroni procedure. *Encyclopedia of research design*, 2010.
