# Supplementary material for: Correction: Independent re-analysis of alleged mind-matter interaction in double-slit experimental data
Source: PLoS One. 2021 Jun 17;16(6):e0253686. doi: 10.1371/journal.pone.0253686 (PMC8211243; doi:10.1371/journal.pone.0253686)
Supplement: S3 File — (PDF) [file pone.0253686.s003.pdf]

Independent re-analysis of alleged mind-matter interaction in double-slit experimental data

Nicolas Tremblay*

CNRS, Univ. Grenoble Alpes, Grenoble INP, GIPSA-lab, Grenoble, France

* nicolas.tremblay@cnrs.fr

Abstract

A two year long experimental dataset in which authors of [1] claim to find evidence of mind-matter interaction is independently re-analyzed. In this experiment, participants are asked to periodically shift their attention towards or away from a double-slit optical apparatus. Shifts in fringe visibility of the interference pattern are monitored and tested against the common sense null hypothesis that such shifts should not correlate with the participant's attention state. We i/ show that the original statistical test used in [1] contains an erroneous trimming procedure leading to uncontrolled false positives and underestimated p-values, ii/ propose a deeper analysis of the dataset, identifying several preprocessing parameters and carefully assessing the results' robustness regarding the choice of these parameters. We observe, as in [1], shifts in fringe visibility in the direction expected by the mind-matter interaction hypothesis. However, these shifts are not deemed significant ($p > 0.05$). Our re-analysis concludes that this particular dataset does not contain evidence of mind-matter interaction.

1 Introduction

The hypothesis of a mind-matter interaction, that is, the possibility that human intention may have an impact on matter at a distance, is usually regarded by most physicists as a highly controversial concept. It is nonetheless related to von Neumann's interpretation [2] of the quantum measurement problem, namely that consciousness causes the collapse of the wave function when a quantum system in a superposition of states is observed. Even if this interpretation has been and still is considered by many minds of quantum mechanics [2–4], it is today blatantly disregarded by a majority of physicists [5] partly because it flirts with the overwhelmingly complex mind/body problem. This mysterious link between consciousness and matter appears indeed to have an infinite number of uncontrollable parameters, and therefore does not seem to lend itself to rigorous scientific inquiry. Moreover, von Neumann's interpretation being by all means only one out of many possible interpretations of quantum mechanics [6] –most of which keep consciousness aside–, physicists generally prefer mathematically controlled objective concepts such as quantum decoherence [7] or Everett's many-worlds interpretation [8]. It is nevertheless well worth reminding that, however strong and heated are personal convictions around this debate, consensus over the quantum measurement problem has not yet been reached [5] and that any attempt to provide empirical information on this matter should be widely welcome.

Along those lines, the experiment first proposed by Ibison and Jeffers in [9] is worthy of interest. Their working hypothesis is that a human subject's attention towards a

quantum system may be modeled as an extremely weak measurement of the system, that should in turn imply a proportionally weak but still *measurable* collapse of its wave function. The authors propose to test this hypothesis using one of the simplest quantum apparatus: the double-slit optical interferometer. In this context, it is well-known [10] that if the path taken by photons through the interferometer (called “which-way information”) is recorded, then photons behave like particles (they don’t interfere), otherwise they behave like waves (they interfere). It has also been verified that the strength of the observed interference pattern is inversely proportional to the amount of which-way information one gathers [11, 12]. Keeping that in mind, and according to the working hypothesis previously stated, a human subject’s attention towards a double-slit system, if it really acts as a weak measurement of the which-way information, should very slightly attenuate the interference pattern. Other working hypotheses can be thought of that do not require a gain in which-way information while still accounting for a decrease in fringe visibility. For instance, Pradhan [14] proposes another theoretical background based on a small modification of the Born rule. We will not delve here into the technicalities of these theoretical approaches and refer to the debates and ideas in [14–17] for the interested reader. In this paper, we will essentially concentrate on data and analyze it as carefully as possible to identify anomalies if they exist, regardless of the precise potential mechanism underlying them.

Ibison and Jeffers reported contradictory and inconclusive results from their pioneering experiments [9]. In the last few years, Radin and collaborators [1, 18, 19] reproduced their experiment at a large scale. In their work, the fringe visibility of the interference pattern is monitored while human subjects are asked to periodically shift their attention towards or away from the optical system. In [1], the authors analyze a two-year long experiment with thousands of subjects, and claim to find small but statistically significant shifts of the fringe visibility, and interpret it as evidence of mind matter interaction. Note that Baer [20] proposed a partial re-analysis of the data and concluded that the data “lead to a possibility, but certainly not a proof, that a psychophysical effect exists” and pointed out that physical noise was too high in the system to draw further conclusions.

In this paper, we independently re-analyze the dataset presented in [1]. We i/ show that the trimming-based[1] statistical procedure used in [1] is flawed and leads to false-positives, as was pointed out to us by Von Stillfried and Walleczek, the authors of a recent article [24] reporting a commissioned replication study of Radin’s double-slit experiment; ii/ provide a bigger picture of the statistical analysis and explore its robustness with respect to several preprocessing choices. As in [1], we observe fringe visibility shifts towards the direction predicted by the mind-matter hypothesis. However, our analysis shows that these shifts are *not* statistically significant, with no p-value under 0.05.

In an effort for reproducible research, the ~ 80 Gb of raw data are publicly available on the Open Science Framework platform at the address `https://osf.io/ywktp/`. Moreover, the Matlab codes used in this paper (and necessary to reproduce all experiments and figures) are available on the author’s website at `http://www.gipsa-lab.fr/~nicolas.tremblay/files/codes_mind_matter.zip`.

The outline of the paper is as follows. We briefly recall the experiment’s protocol in Section 2.1, and define the difference in fringe visibility $\Delta\nu$ in Section 2.2 as the main statistics we will focus our analysis on. Sections 2.3 and 2.4 detail the basic statistical tests we perform and preliminary results. The robustness of these results is then assessed in the subsequent Sections 2.5 to 2.8. Section 3 discusses all theses analyses and compares them to the results originally obtained by Radin et al. [1]. Section 4

[1] trimming removes a given percentage of the lowest and the highest values in the dataset, and is used to remove possible outliers from the data

offers concluding remarks.

2 Materials and methods

2.1 The experiment

The apparatus consists of a laser, a double-slit, and a camera recording the interference pattern; and is located in IONS' laboratory, in Petaluma, California. Details are in [1]. The apparatus is always running, even though the data is only recorded when somebody connects to the system via Internet. A participant to the experiment connects online to the server (accessible through IONS' research website) and receives alternating instructions every 30 seconds, to either "now concentrate" or "now relax". During concentration epochs, the participant's task is to mentally influence the optical system in order to increase a real-time feedback signal, displayed as a dynamic line on the screen. For people who prefer to close their eyes during the experiment, the feedback is also transmitted as a whistling wind tone.

In 2013, the feedback was inversely proportional to a sliding 3-second span average of the fringe visibility: the higher the line, or the higher the pitch of the tone, the lower was the fringe visibility, the closer was the system to "particle-like" behaviour.

In 2014, due to a coding error, the feedback was inversed: the feedback now increased when the fringe visibility *increased*. The participant's task was still to increase the feedback, but this time the higher the line, or the higher the pitch of the tone, the lower was the fringe visibility, the closer was the system to "wave-like" behaviour.

As controls, a Linux machine connects to the server via Internet at regular intervals. The server does not know who it is dealing with: it computes and sends feedback, and records interference data just as it would do for a human participant.

Each session always starts and finishes with a relaxation epoch. A total of 10 concentration and 11 relaxation epochs are recorded per session, which makes the whole session last about 10 minutes and 30 seconds. Some sessions end before all epochs are completed, due to Internet connection issues, or to participants' impatience. One possible bias could come from participants' self-selection: it could be argued that participants with poor results quit the experiment earlier than participants performing well. To avoid this bias, we need to take as many sessions as possible into account. On the other hand, very short sessions do not enable a precise estimation of any measurable difference between the two types of epochs. We decide to keep only sessions containing more than $\tau = 1000$ camera frames, which correspond to sessions approximately completed half-way and containing 8 alternating epochs. We will see in Section 2.7 how the value of τ changes the results.

Given $\tau = 1000$, the dataset is comprised of 3679 sessions in 2013 (2374 of which are controls) and 4976 in 2014 (3363 of which are controls).

2.2 Pre-analysis: from the raw data to difference in fringe visibility

The camera records at 4Hz a line of 3000 pixels, an example of which is shown in Fig 1, where are also displayed the maximum (noted env_M) and minimum (note env_m) envelopes of the interference pattern computed with cubic spline interpolation between local extrema. Local extrema are automatically detected after a Savitzky-Golay filter of order 2 on a 29-pixel moving window that smooths the interference pattern in order to remove the pixel jitter that appears on some camera frames. We have also tried other smoothing options: same order Savitzky-Golay filters with 39 and 49-pixel

window-lengths, as well as simple moving average filters with 20 and 30-pixel window-lengths, with no significant change in the overall results.

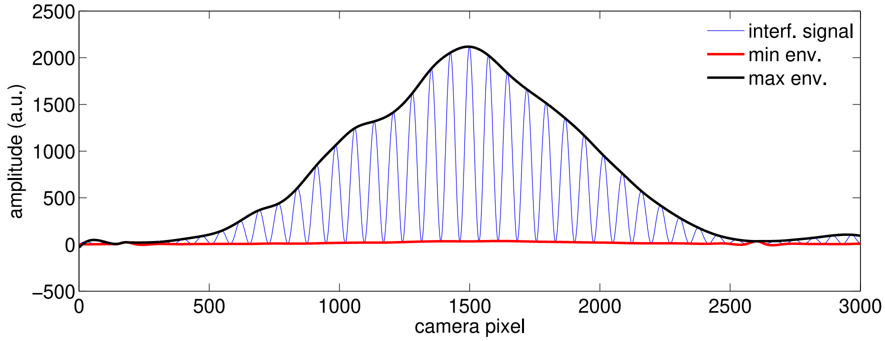

Fig 1. The interference pattern. Example of a camera shot of the interference pattern, along with its two spline interpolated envelopes.

For a better signal to noise ratio, we consider the 19 middle fringes of the pattern. Fig 2 shows such a zoom, as well as the fringe visibility function, defined as:

$$fv = \frac{\text{env}_M - \text{env}_m}{\text{env}_M + \text{env}_m}. \tag{1}$$

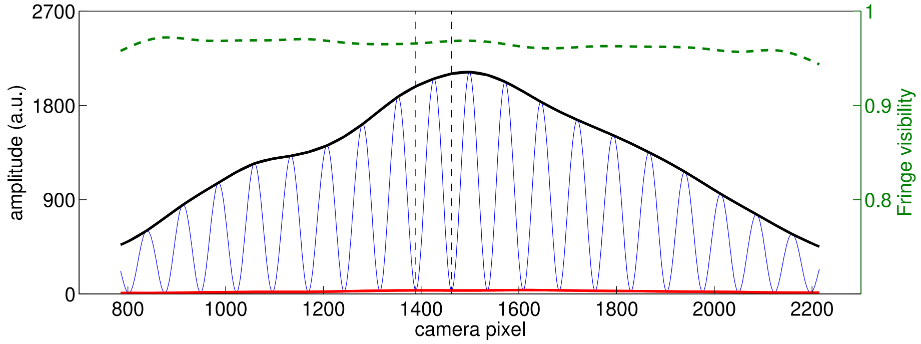

Fig 2. Zoom on the interference pattern. Zoom around the 19 middle fringes of the interference pattern, along with its two interpolated envelopes. The fringe visibility as defined in Eq 1 is represented by the dashed green line. The two vertical dashed lines represent the interval corresponding to fringe number 9.

For each camera frame, we extract one scalar. The choice of this scalar is not straightforward and we will explore different choices throughout the paper. Following the analyses published in [1], we start by concentrating on the average of the fringe visibility around fringe number 9, that is, on the interval represented in Fig 2 between two vertical dashed lines. We will see in Section 2.5 how results change if one considers other fringe numbers, or averages over more than one fringe.

Fig 3 shows fringe 9's visibility versus time during one typical session. The epochs, as sent by the server, are represented with the square signal: high values represent relaxation epochs, and low values concentration epochs.

For each session, we extract a single scalar value: the difference between the median of the fringe visibility during concentration epochs, and the median of the fringe visibility during relaxation epochs. The medians are considered as they are more robust to outliers than the average. Formally, given the fringe visibility time series fv, define

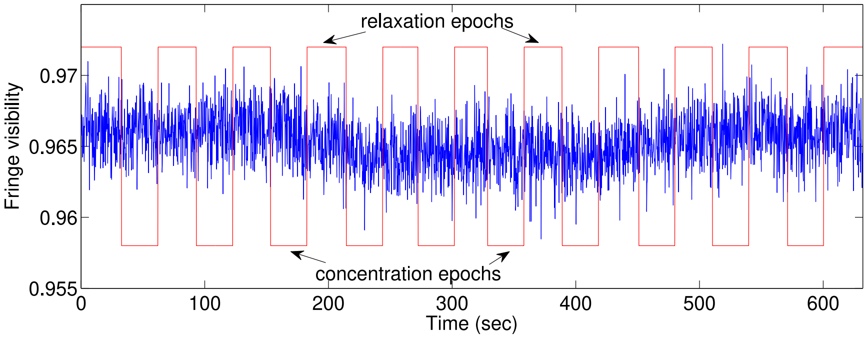

Fig 3. Fringe 9's visibility versus time for a typical session. The red square signal represents the concentration/relaxation epochs.

fv^c (resp. fv^r) as the reduction of fv to the concentration (resp. relaxation) epochs, and $\Delta\nu$ as the difference in median fringe visibility:

$$\Delta\nu = \text{median}(fv^c) - \text{median}(fv^r) \in \mathbb{R}. \quad (2)$$

$\Delta\nu$ is the statistics we will use in the following analyses.

2.3 Zero mean statistical testing

If the mind-matter interaction hypothesis is false, one would normally expect $\mathbb{E}(\Delta\nu)$ to be equal to zero. Denote by $\mathcal{X}$ the set to test (for instance, it could be the set of all values of $\Delta\nu$ measured across all human sessions in 2013) and denote by n its size. We test the zero-mean hypothesis, denoted by H_0, by performing a trimmed mean percentile bootstrap test (following Section 4.4.4 of [13]). Let $0 \le q \le 1$ be the intensity of the trim. Let B be the number of bootstraps (we use $B = 5 \times 10^4$ in our experiments). The statistical procedure is the following:

1. Generate a bootstrap sample $\mathcal{X}_1^*$ by sampling uniformly with replacement n elements of $\mathcal{X}$.

2. Trim the bootstrap sample: denoting by r_q the integer closest to $qn/2$, remove the r_q lowest and r_q highest values from $\mathcal{X}_1^*$, obtaining $\mathcal{X}_{1,q}^*$ of size $n - 2r_q$.

3. Compute the sample mean $\bar{x}_{1,q}^*$ of $\mathcal{X}_{1,q}^*$.

4. Repeating steps 1 to 3 B times yields B bootstrap trimmed sample means: $\mathcal{B} = \{\bar{x}_{1,q}^*, \ldots, \bar{x}_{B,q}^*\}$.

5. Consider $0 \le \alpha \le 1$ a chosen significance level. Let $l = \alpha B/2$, rounded to the nearest integer, and let $u = B - l$. Letting $\bar{x}_{(1),q}^* \le \ldots \le \bar{x}_{(B),q}^*$ represent the B bootstrap estimates in ascending order, a $1 - \alpha$ confidence interval for the underlying mean is: $\left(\bar{x}_{(l+1),q}^*, \bar{x}_{(u),q}^*\right)$. If 0 is not in this interval, H_0 is rejected with significance level α. The probability that a bootstrap trimmed sample mean verifies $\bar{x}_q^* < 0$ is readily estimated by A/B, where A is the number of bootstrap samples whose trimmed sample mean is inferior to 0. The associated p-value is thus estimated by $p = 2\min\left(\frac{A}{B}, 1 - \frac{A}{B}\right)$.

Output: - p a p-value
- (*optional*) a normalized shift $\frac{\bar{\mathcal{B}}}{\text{std}(\mathcal{B})}$, where $\bar{\mathcal{B}}$ (resp. $\text{std}(\mathcal{B})$) is the sample mean (resp. sample standard deviation) of the bootstrap set $\mathcal{B}$.

Note that this normalized shift is only computed for illustration purposes (in order to observe in which direction potential shifts of the mean appear): it is *not* used for the statistical test. Also, note that in the study by Radin et al. [1], the trimming is performed *before* generating the bootstrap samples (steps 1 and 2 are inverted), which creates false positives as soon as $q > 0$, as illustrated in the Supporting information. In this first analysis, q is set to 20%. We will see later in Section 2.6 how this choice affects the results.

A time lag l is expected between the fringe visibility and the alternating instructions of concentration and relaxation. Indeed, a lag could occur for three main reasons: first due to the time needed to switch one's attention from a concentration state to another, second due to the finite (and possibly slow) speed of the Internet connection, and third due to the 3 seconds span of the sliding window on which the feedback is computed. In the following, we will consider lags between 0 and 25 seconds.

The null hypothesis we are testing is therefore: H_0*: considering any time lag,* $\mathbb{E}(\Delta\nu)$ *is null.* Indeed, common sense suggests that whatever the concentration state of a participant, there is no reason that the fringe visibility of the optical system should be affected. This hypothesis involves multiple testing ($m = 26$ tests precisely): one for each time lag l. For each time lag l we test the null hypothesis: H_0^l*: considering time lag* l*,* $\mathbb{E}(\Delta\nu)$ *is null*, that will output a p-value p_l. We then apply the Holm-Bonferonni method [22] to adjust for multiple comparison, and obtain an overall p-value p^{H_0} for H_0. To this end, write $p_{(1)} \leq p_{(2)} \leq \ldots \leq p_{(m)}$ the values of $\{p_l\}$ sorted in ascending order. The overall p-value p^{H_0} is then formally defined as:

$$p^{H_0} = \min_{k=1,2,\ldots,m} (m - k + 1)\, p_{(k)}. \tag{3}$$

This method is regarded as pessimistic in our context of correlated tests [23]. But in this controversial field of research, it is safer to use pessimistic estimations.

2.4 Preliminary results and remarks

Fig 4 shows the normalized shift and p_l versus the time lag l, for the human and control sessions of each year. The corrected p-value for multiple comparisons corresponding to H_0 for the human '13 sessions (resp. control '13, human '14, control '14) is $p^{H_0} = 7 \times 10^{-2}$ (resp. 1, 1, 1). These values call for a few preliminary observations. As in [1], we find that both years' control data act as expected by H_0. We also observe a shift towards negative values for the 2013 human sessions, even though in a much less significant manner than in [1]. Finally, we observe a shift towards positive values for the 2014 human sessions, but it is deemed insignificant.

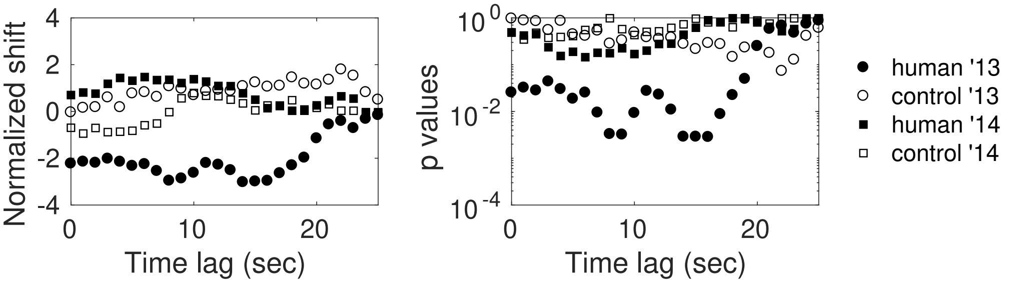

Fig 4. Result of the zero-mean test versus the time lag for fringe 9. Normalized shift and p-values (corresponding to hypotheses H_0^l) versus the time lag, for the human and control sessions of each year. Results are shown for $q = 0.2$, $\tau = 1000$.

We now propose to make a very different choice in the analysis of this data than the one originally proposed. The authors in [1] propose to aggregate the data from both

years, after inverting the sign of the 2014's $\Delta\nu$ values to account for the feedback's accidental sign inversion. We argue in this paper that aggregating the data is confusing and makes results' interpretation more difficult. In this preliminary analysis, 2014's data slightly shift towards positive values, but within chance expectations. Given that there was no reason to believe before the experiment that such a positive shift would be observed, one could argue that aggregating the data after a sign inversion is using a possibly random fluctuation to one's advantage. Another possibility is to aggregate the data without the sign inversion. This is not reasonable given the fact that experimental conditions (specifically the feedback, which seems to be very important) were different for both years. The most reasonable decision regarding both years' analyses is to keep them separate – at the cost of lower statistical power.

Another fundamental difference between our analysis and the one proposed in [1] is prior knowledge regarding the time lag to consider. Authors in [1] build upon their previous (and independent) experiment [19] that indicated a time lag of 9 seconds as a good parameter to discriminate humans from controls (as long as the experiment used to learn this parameter and the experiment used to test this parameter are independent, this is perfectly possible). In our independent re-analysis, we prefer the safer choice of no prior knowledge, thereby necessarily testing several time lags followed by constraining adjustments due to multiple testing – at the cost, once again, of lower statistical power.

Note that, for the sake of completeness, we will later show (in Figure 12 with the discussion in Section 3) the results obtained by aggregating both years' data after sign inversion and/or supposing prior knowledge of the time lag. For now, however, we keep both years' data separate, and test against several time lags.

In the next four sections (Section 2.5 to Section 2.8), we look at the robustness of the results regarding all the seemingly arbitrary decisions we made at every step of this pre-analysis, namely: the fringe number to consider (we chose fringe 9), the trimming intensity q (we chose $q = 20\%$), the length threshold τ under which we deem sessions too short to give any reasonable estimation of $\Delta\nu$ (we chose $\tau = 1000$ camera frames), and fv's estimation method (we chose the normalized difference between spline interpolated envelopes).

2.5 Extending the analysis to all fringes

Fringe number 9 is an arbitrary choice and it is necessary to look at other fringes. Fig 5 shows results obtained for fringe number 7: the shifts observed for the human sessions are in the same direction than for fringe number 9, with a less (resp. more) significant result for 2013 (resp. 2014) with a corrected p-value of $p^{H_0} = 3 \times 10^{-1}$ (resp. 6×10^{-1}). The big surprise comes from the 2013 control sessions that show a significant ($p^{H_0} = 7 \times 10^{-3}$) increase of $\Delta\nu$. Once again, this is different from the results shown in Fig 2 of [1] where the 2013 controls are within chance expectation for all fringes. This is mainly due to the combination of two facts: i/ they suppose a prior knowledge of a 9 second time lag and we do not, and ii/ large anomalies of the 2013 control data occur after 9 seconds – see Fig 5.

To look at all fringes at once, Fig 6 shows the corrected p-values p^{H_0} as a function of the fringe number for all four different session types. We see how a particular choice of fringe for the analysis is problematic: depending on this choice one may serve different outcomes of the statistical test! For instance, one could p-hack and choose *a posteriori* fringe number 14 as a good candidate to discriminate humans from controls; or choose fringe number 19 to conclude that one cannot discriminate one from the other.

To go further, and in order to prevent us from choosing the fringe number(s) that serve one hypothesis or the other, we propose two strategies that both take into account information from all fringes.

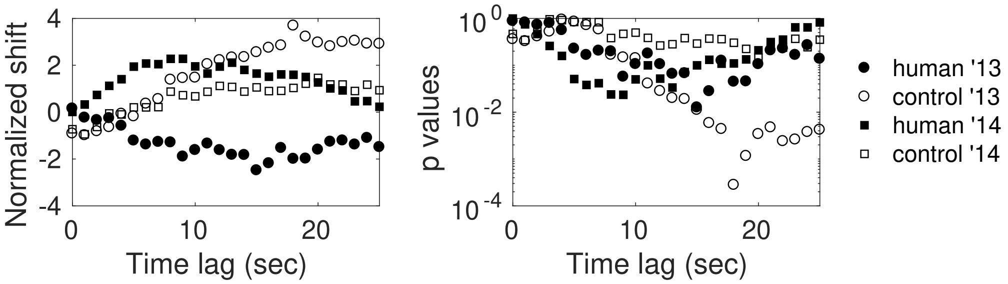

Fig 5. Result of the zero-mean test versus the time lag for fringe 7. Normalized shift and p-values (corresponding to hypotheses H_0^l) versus the time lag, for the human and control sessions of each year. Results are shown for $q = 0.2$, $\tau = 1000$.

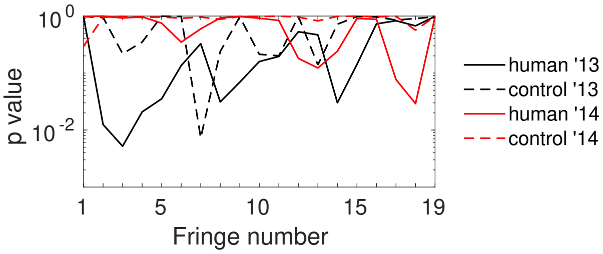

Fig 6. Corrected for multiple comparisons p-values corresponding to hypothesis $\mathbf{H_0}$ for the human and control sessions of each year as a function of the fringe number. Results are shown for $q = 0.2$ and $\tau = 1000$.

A new null hypothesis. We propose to investigate a new null hypothesis comprehending all fringes: H_0': *considering any time lag and any fringe number,* $\mathbb{E}(\Delta\nu)$ *is null.* Testing H_0' implies doing $m' = 26 \times 19 = 494$ individual tests (26 time lags for each of the 19 fringes). We correct for multiple comparisons using the same Holm-Bonferonni method that becomes even more conservative given that we add many correlated tests. Keeping that in mind, we obtain a corrected p-value for the 2013 human (resp. 2013 control, 2014 human, 2014 control) sessions of $p^{H_0'} = 10^{-1}$ (resp. 10^{-1}, 5×10^{-1}, 1). Fig 7 shows the normalized shift of each of the 494 individual tests versus the time lag and the fringe number: the direction from which the data differs from the null hypothesis H_0^l is consistent across all individual tests. The 2013 (resp. 2014) human sessions show a negative (resp. positive) shift. The 2013 control sessions show a positive shift while the 2014 control sessions do not show a consistent shift. These shifts are however not deemed significant.

A new fringe visibility definition. The variability observed in Fig 6 could be due to a signal-to-noise ratio (SNR) that is too small for our task. In order to increase the SNR, we define $\overline{fv}_\mu$ the average of fv over all fringes between $10 - \mu$ and $10 + \mu$ (with μ an integer between 0 and 9). We choose to concentrate on intervals centered around fringe 10 as it is the one with the best SNR. We could of course choose other intervals to average over but we would encounter the very same problem we are trying to avoid: different intervals will serve different hypotheses and a particular choice of interval would be difficult to justify. Here, we rely on the (strong) SNR argument to choose to look at all intervals centered around fringe 10.

Given this new definition of fringe visibility, we test the null hypothesis: H_0'': *considering any time lag and any* μ, $\mathbb{E}(\Delta\nu)$ *is null.* Testing H_0'' implies doing $m'' = 26 \times 10 = 260$ individual tests (26 time lags for each of the 10 possible choices for μ). After correction for multiple comparisons, we obtain a corrected p-value for the 2013

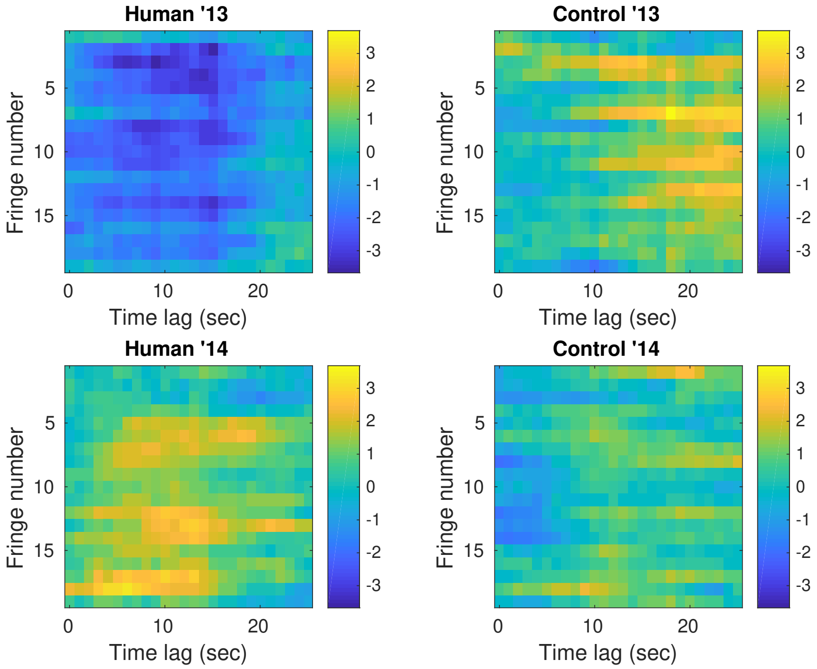

Fig 7. Normalized shifts of all tests performed in H_0'. Normalized shifts of each of the 494 individual tests versus the time lag and the fringe number for all four different types of sessions. Results are shown for $q = 0.2$ and $\tau = 1000$.

human (resp. 2013 control, 2014 human, 2014 control) sessions of $p^{H_0''} = 10^{-1}$ (resp. 1, 1, 1). Fig 8 shows the normalized shift of each of the 260 individual tests versus the time lag and μ: the direction of the observed shifts is the same as previously.

Summary. We first observed that results are not robust with respect to the choice of fringe number one studies. To avoid choosing a fringe number, we i/ performed a test whose null hypothesis encompasses all fringe numbers, ii/ performed a test on the average of the fringe visibility over central fringes. Both analyses show the following:

- the 2013 human sessions shift towards negative $\Delta\nu$ values;
- the 2014 human sessions shift towards positive $\Delta\nu$ values;
- the 2013 control sessions shift towards positive $\Delta\nu$ values;
- the 2014 control sessions do not show a clear and consistent shift;
- all these shifts are however deemed insignificant ($p > 5 \times 10^{-2}$) after correcting for multiple testing.

We now investigate if these results are robust to i/ the trimming intensity q in Section 2.6, ii/ the length threshold τ in Section 2.7, iii/ the fringe visibility estimation method in Section 2.8.

2.6 Robustness regarding the trimming intensity q

Fig 9 shows the p-values $p^{H_0'}$ and $p^{H_0''}$ for the four different types of sessions versus the the trimming intensity q. The direction of the shifts (not shown) do not change and are as previously stated. They are not deemed significant for any choice of q: results as summarized at the end of Section 2.5 are robust with respect to q.

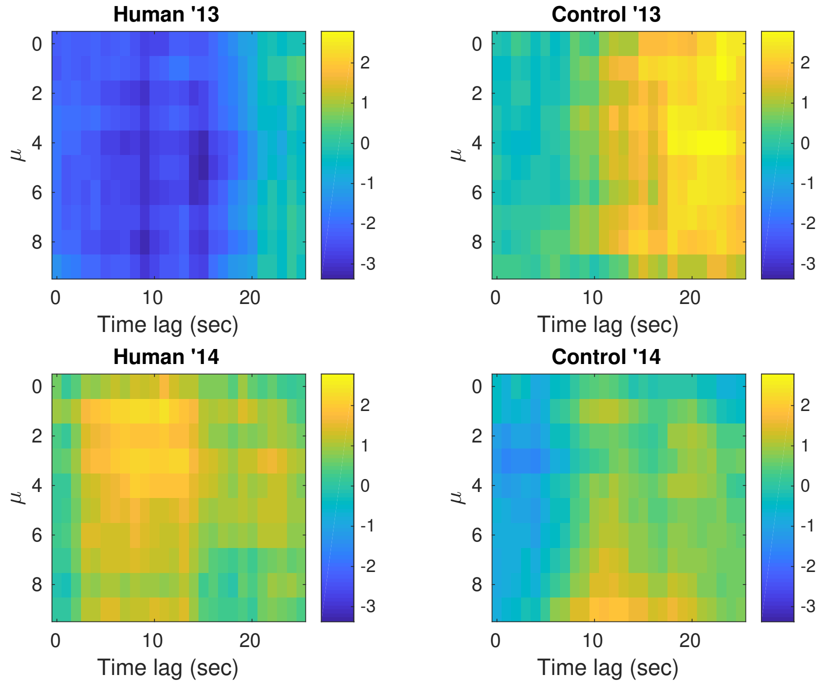

Fig 8. Normalized shifts of all tests performed in $\mathbf{H_0}$". Normalized shifts of each of the 260 individual tests versus the time lag and μ for all four different types of sessions. Results are shown for $q = 0.2$ and $\tau = 1000$.

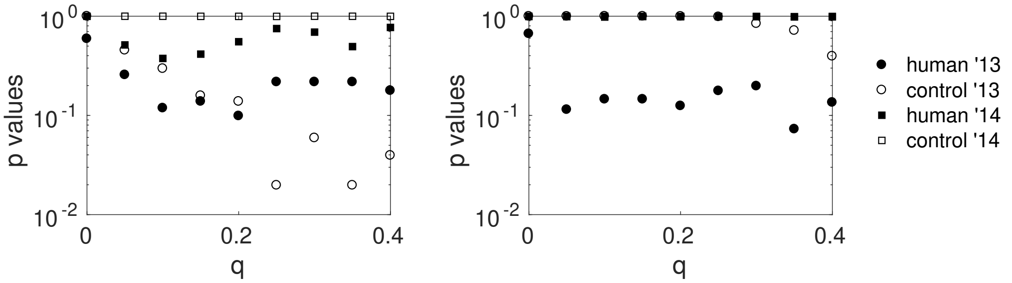

Fig 9. Robustness regarding the trimming intensity q. Corrected for multiple comparisons p-values corresponding to H_0' (left) and H_0'' (right) for the four types of sessions as a function of the trimming intensity q. Results are shown for $\tau = 1000$.

2.7 Robustness regarding the length threshold τ

We recall that τ is the threshold under which we deem sessions too short to estimate $\Delta\nu$ correctly. Fig 10 shows the p-values $p^{H_0'}$ and $p^{H_0''}$ versus q for two other values of τ: the results are robust regarding the length threshold. In the following, we consider only results obtained with $\tau = 1000$.

2.8 Robustness regarding the fringe visibility estimation method

Until now we have been using the normalized difference between the interpolated envelopes as the definition of the fringe visibility (see Eq (1)). It is necessary to look at the sensitivity of the results with regards to that method of estimation. Authors in [1] define the visibility of fringe n as the normalized difference between the n-th local

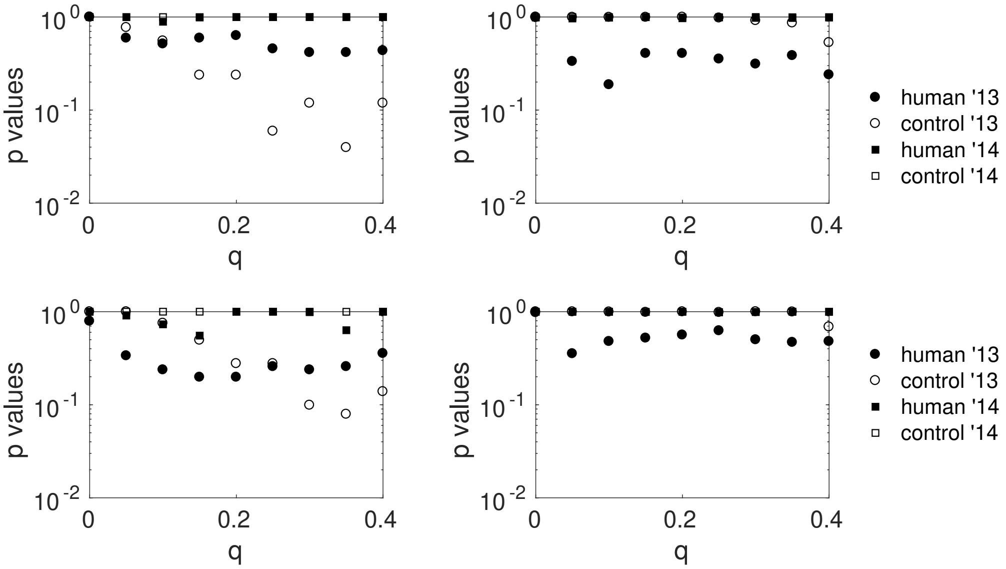

Fig 10. Robustness regarding the session length threshold τ. Corrected for multiple comparisons p-values corresponding to H_0' (left) and H_0'' (right) for the four types of sessions as a function of the trimming intensity q, for length thresholds $\tau = 1700$ (top) and $\tau = 2300$ (bottom).

maximum M_n and its preceding local minimum m_n:

$$fv = \frac{M_n - m_n}{M_n + m_n}. \quad (4)$$

Results obtained with this definition on fringe 9, and with $q = 20\%$ and $\tau = 1000$, are shown in Fig 11 (top). We observe significant anomalies (even though much less significant than in [1]) in the human data of both years especially around $l = 9$ seconds, and insignificant results for the controls. Fig 11 (middle) gives the bigger (and corrected for multiple comparisons of the time lag) picture by plotting the p-value p^{H_0} for the four types of sessions versus the fringe number. Once again, depending on the fringe one considers, one may be lead to contradictory conclusions. One therefore needs to consider hypotheses H_0' and H_0''. Fig 11 (bottom) shows the p-values $p^{H_0'}$ and $p^{H_0''}$ versus the trimming intensity q: all p-values are larger than 5×10^{-2}.

For a fringe number n and its associated local maximum M_n, there is no reason to define its visibility by comparing M_n to its previous local minimum m_n rather than its succeeding local minimum m_{n+1}. If one defines

$$fv = \frac{M_n - m_{n+1}}{M_n + m_{n+1}}, \quad (5)$$

then one obtains similar results (not shown).

One concludes that the results as summarized at the end of Section 2.5 are robust with respect to the fringe visibility estimation method.

3 Discussion

The preliminary analysis proposed in Section 2.4 is subject to four seemingly arbitrary choices: the fringe number, the minimal length of a session, the trimming intensity q and the choice of the fringe visibility estimation method. In Section 2.5, we observe that

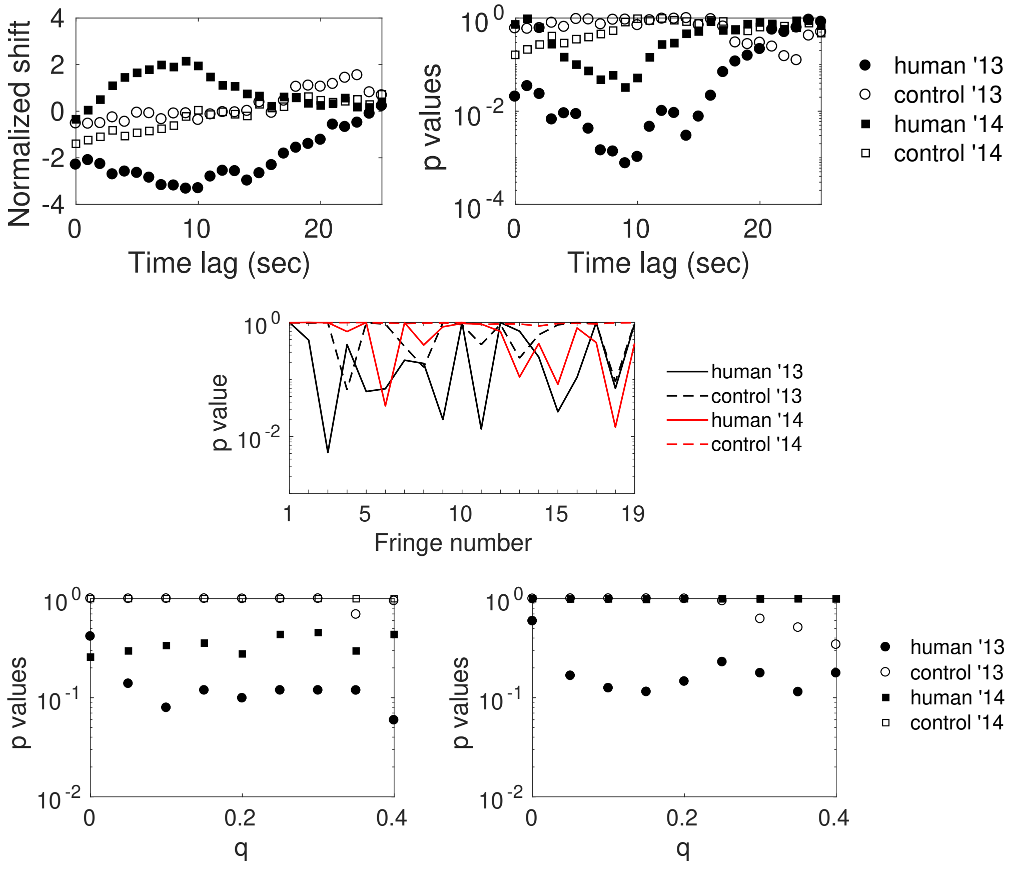

Fig 11. Test results using Eq 4 to define the fringe visibility. (top) Normalized shifts and p-values p_l versus the time lag for fringe number 9 (with $q = 20\%$), (middle) p-value p^{H_0} versus the fringe number (with $q = 20\%$) and (bottom) p-value $p^{H_0'}$ (left) and $p^{H_0''}$ (right) versus the trimming intensity.

different fringe choices change the output of the statistical tests, and thus the conclusions that may be drawn from the data. We therefore propose two more robust methods that avoid choosing fringes: the first one is to encompass all fringes in the null hypothesis, leading to H_0', and the second is to average the fringe visibility over central intervals of fringes, leading to H_0''. Both null hypotheses lead to the following observations:

- the 2013 human sessions shift towards negative $\Delta\nu$ values;
- the 2014 human sessions shift towards positive $\Delta\nu$ values;
- the 2013 control sessions shift towards positive $\Delta\nu$ values;
- the 2014 control sessions do not show a clear and consistent shift;
- all these shifts are deemed insignificant ($p > 5 \times 10^{-2}$) after correcting for multiple testing.

We show that these results are robust regarding the intensity q of the trim in Section 2.6, the minimal session length in Section 2.7, and the fringe visibility estimation method in Section 2.8.

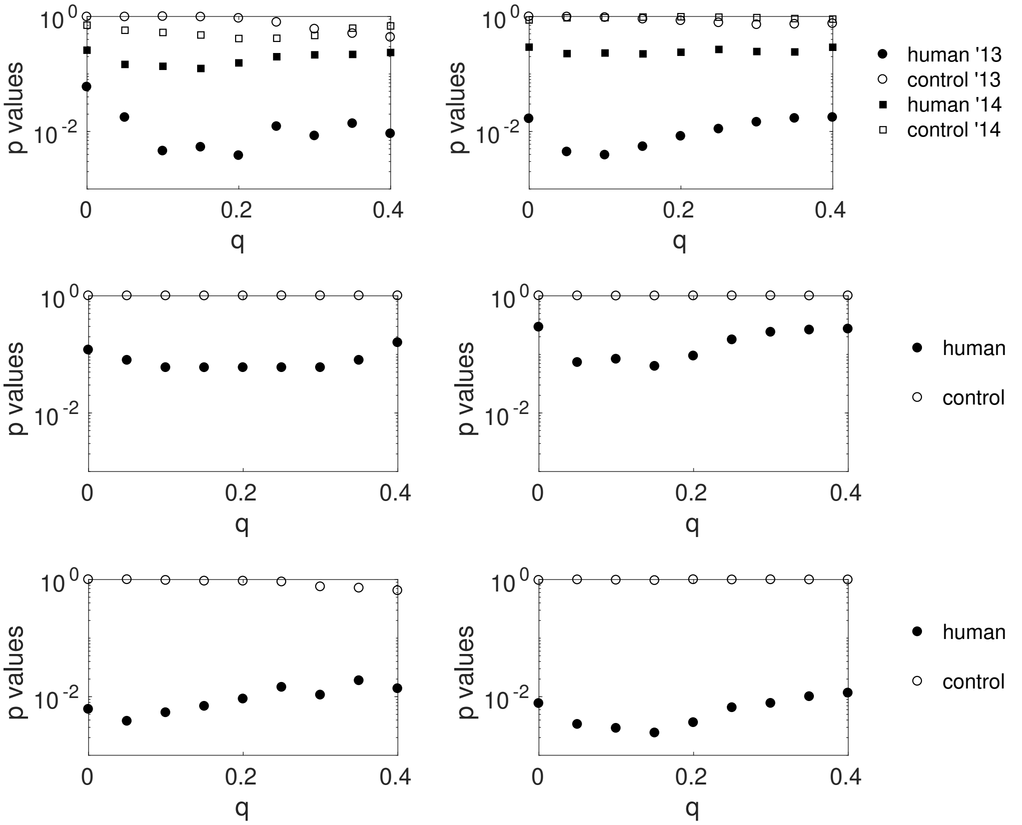

Fig 12. Results one would have obtained instead of Figure 9 in the following three scenarios. (top) Scenario 1: a time lag of 9 seconds is chosen from the start, and both years are analyzed separately. (middle) Scenario 2: 26 different time lags are tested and then corrected for multiple comparisons, and the data from both years are combined after sign inversion for 2014. (bottom) Scenario 3: a time lag of 9 seconds is chosen from the start, and the data from both years are combined after sign inversion for 2014.

Comparison with results in [1] In the original paper reporting this experiment [1], the authors report that "the results showed that with human observers the fringe visibility at the center of the interference pattern deviated from a null effect by 5.72 sigma ($p = 1.05 \times 10^{-8}$), with the direction of the deviation conforming to the observers' intentions." Such a small p-value is obtained by the authors for three main reasons: i/ the trimming procedure they used is erroneous (trimming should be done after bootstrapping, not before) and outputs underestimated p-values (possibly of several orders of magnitude) as soon as q is strictly superior to 0, as illustrated in the Supporting information, ii/ the sign of the 2014 data is reversed to account for the accidental sign inversion of the feedback and the analysis is then performed on all data combined: combining the 2013 data with the sign reversed 2014 data, iii/ a lag of 9 seconds is chosen from the start based on a previous (and independent) experiment [19] that indicated that such a time lag was a good parameter to discriminate humans from controls.

In this paper, we corrected point i/ and we argued that points ii/ and iii/ were not solid choices from our statistical re-analysis point-of-view, and preferred a more conservative standpoint by analyzing both years separately and testing 26 different time

lags before correcting for multiple comparisons; both these choices necessarily inducing a lower statistical power. For completeness, we show in Figure 12 the results one would have obtained instead of Fig. 9 in three different scenarios, in which we set the time lag at 9 seconds from the start and/or combine both years after sign inversion for 2014. We observe that the results look more convincing in these scenarios, with large p-values (> 0.7) for the controls, and slightly significant deviations for the humans. However, all p-values in these three scenarios are larger than 2×10^{-3}: they cannot be interpreted as strong evidence of mind-matter interaction, but may motivate further replication attempts. These additional results seem to point out that the erroneous statistical test used in [1] lead to an underestimation of the p-value by 5 orders of magnitude (they reported a p-value of $\sim 10^{-8}$ instead of the $\sim 10^{-3}$ that we find here) –which further lead the authors to erroneous conclusions.

Before we conclude, let us make an important statement. We have made many statistical tests, and to prevent p-hacking, one needs to look at all these tests as a whole. Extracting one test or the other from the whole is not recommended. Note that, on top of the tests discussed in the paper we have also performed tests with two other fringe visibility definitions: the average of Eqs (4) and (5), and the fringe visibility extracted by spline interpolation as in Eq (1) but sampled only at the extrema instead of considering the average over each fringe as presented here. None of these tests showed a significant difference than the ones shown in the paper.

4 Conclusion

The thorough analysis pursued in this paper contradicts the results previously published in [1]. On the one hand, we observe shifts of the fringe visibility in the direction predicted by the mind-matter interaction hypothesis, as in [1]. On the other hand, these shifts are not deemed significant by our analysis.

Supporting information

Let $0 < \alpha < 1$ be a significance level. We illustrate here that false-positives are uncontrolled in the test used in [1] and under control of α in the correct test described in Section 2.3. To do so, consider the following framework:

i/ Create a synthetic set $\mathcal{X}$ by drawing its n iid elements from $\mathcal{N}(0,1)$, the Gaussian distribution with zero mean and variance 1. n is set to 10^3.

ii/ Generate $N = 5 \times 10^4$ independent realisations of such set $\mathcal{X}$. All N sets thus have a true zero mean by construction.

iii/ Test each set: obtain a p-value per set and, given the significance level α, a rejection decision per set.

We then consider both the probability of type I error estimated by $\hat{p}_{\mathrm{I}} = R/N$, where R counts the number of rejected sets $\mathcal{X}$ (for the given α), as well as the α-quantile p^*_α of the N p-values obtained: the value under which there are αN p-values. If the test used in iii/ is correct, both $\hat{p}_{\mathrm{I}}$ and p^*_α should be very close to α.

Figure 13 compares results obtained with the test published in [1] and the one described in Section 2.3. For both tests, at $q = 0$ and as expected, both indicators $\hat{p}_{\mathrm{I}}$ and p^*_α are at α, as it should be. However, as q increases, the test from [1] deviates from α quite significantly. For instance, for $q = 0.2$, the 0.01-quantile of the computed p-values is 3.5×10^{-4}, almost two orders of magnitude lower than what it should be!

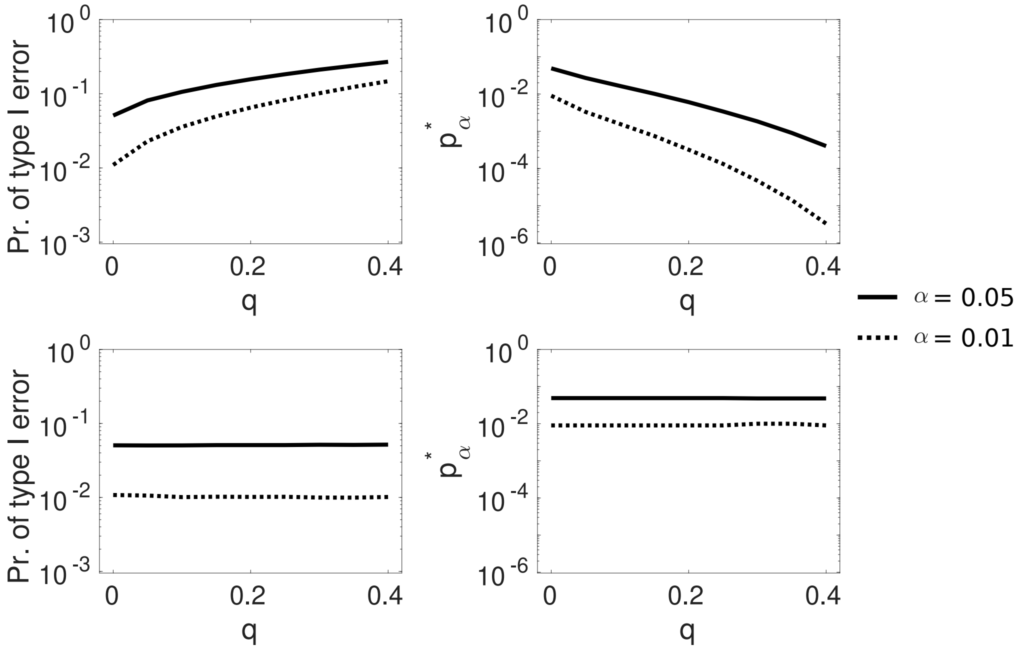

Fig 13. Results obtained on artificial data of zero mean (see the Supporting information for details). Top line: results of the test published in [1]. Bottom line: results of the correct test detailed in Section 2.3. Left: the estimated type I error $\hat{p}_{\mathrm{I}}$ as a function of q (the trimming intensity), for two different values of α. Right: the α-quantiles of the p-values versus q, for two different values of α. Number of bootstrap samples used for both tests: 2000.

On the contrary, in the test used in this paper, and for all values of q, both $\hat{p}_{\mathrm{I}}$ and p^*_α are equal to what is expected from a well-controlled test, namely α.

Acknowledgments

We would like to thank the authors of [1] for an unlimited access to both years' data, and their patience in answering all our questions regarding technical details of the data and the experimental protocol. Also, we thank Nikolaus von Stillfried and Jan Walleczek (Phenoscience Laboratories, Berlin) for pointing out to us a statistical error in the original version of this manuscript. They identified the issue in the context of a research project sponsored by the Fetzer Franklin Fund of the John E. Fetzer Memorial Trust.

References

1. D. Radin, L. Michel and A. Delorme Psychophysical modulation of fringe visibility in a distant double-slit optical system. *Physics Essays*, vol. 29, number 1, pp 14–22, 2016.

2. J. Von Neumann, Mathematical foundations of quantum mechanics. *Princeton university press*. 1955.

3. E. Wigner and H. Margenau, Symmetries and reflections, scientific essays. *American Journal of Physics*, vol 35, number 12, pp 1169–1170, 1967.

4. H. Stapp, Quantum theory and the role of mind in nature. *Foundations of Physics*, vol 31, number 10, pp. 1465–1499, 2001.

5. M. Schlosshauer, J. Kofler and A. Zeilinger, A snapshot of foundational attitudes toward quantum mechanics. *Studies in History and Philosophy of Science Part B: Studies in History and Philosophy of Modern Physics*, vol 44, number 3, pp 222–230, 2013.

6. M. Jammer, The Philosophy of Quantum Mechanics: The Interpretations of Quantum Mechanics in Historical Perspectives. *John Wiley*, 1974.

7. M. Schlosshauer, Decoherence, the measurement problem, and interpretations of quantum mechanics. *Reviews of Modern Physics*, vol 76, number 4, 2005.

8. H. Everett, "Relative state" formulation of quantum mechanics. *Reviews of modern physics*, vol 29, number 3, 1957.

9. M. Ibison, and S. Jeffers, A double-slit diffraction experiment to investigate claims of consciousness-related anomalies. *Journal of Scientific Exploration*, vol 12, pp. 543–550, 1998.

10. R. Feynman, The Feynman Lectures on Physics. Volume III: Quantum Mechanics. *Addison-Wesley*, 1966.

11. B. Englert, Fringe visibility and which-way information: An inequality. *Physical review letters*, vol 77, number 11, 1996.

12. S. Dürr, T. Nonn and G. Rempe, Fringe visibility and which-way information in an atom interferometer. *Physical review letters*, vol 81, number 26, 1998.

13. Rand R. Wilcox. Introduction to robust estimation and hypothesis testing. *Academic press*, 2011.

14. R. Pradhan, An explanation of psychophysical interactions in the quantum double-slit experiment. *Physics Essays*, vol. 28, number 3, pp 324–330, 2015.

15. R. Pradhan, Psychophysical Interpretation of Quantum Theory. *NeuroQuantology*, vol. 10, number 4, pp 629–646, 2012.

16. M. Sassoli de Bianchi, Quantum measurements are physical processes. Comment on" Consciousness and the double-slit interference pattern: Six experiments," by Dean Radin et al.[Phys. Essays 25, 157 (2012)]. *Physics Essays*, vol. 26, number 1, pp 15–20, 2013.

17. F. Pallikari, On the question of wavefunction collapse in a double-slit diffraction experiment. *arXiv*, 1210.0432, 2012.

18. D. Radin, L. Michel, K. Galdamez, P. Wendland, R. Rickenbach and A. Delorme, Consciousness and the double-slit interference pattern: Six experiments. *Physics Essays*, vol. 25, number 2, pp 157–171, 2012.

19. D. Radin, L. Michel, J. Johnston and A. Delorme, Psychophysical interactions with a double-slit interference pattern. *Physics Essays*, vol. 26, number 4, pp 553–566, 2013.

20. W. Baer, Independent verification of psychophysical interactions with a double-slit interference pattern. *Physics Essays*, vol 8, number 1, pp. 47–54, 2015.

21. J. Osborne and A. Overbay, The power of outliers (and why researchers should always check for them). *Practical assessment, research & evaluation*, vol 9, number 6, 2004.

22. S. Holm, A simple sequentially rejective multiple test procedure. *Scandinavian journal of statistics*, pp. 65–70, 1979.

23. H. Abdi, Holm's sequential Bonferroni procedure. *Encyclopedia of research design*, 2010.

24. J. Walleczek, N. von Stillfried. False-Positive Effect in the Radin Double-Slit Experiment on Observer Consciousness as Determined With the Advanced Meta-Experimental Protocol. *Frontiers in Psychology*, vol. 10, 2019.
